# Synthetic Approaches Toward Phosphorus-Containing BODIPY and Squaraine Dyes: Enhancing Versatility of Small-Molecule Fluorophores

**DOI:** 10.3390/molecules30010116

**Published:** 2024-12-31

**Authors:** Jeanne M. Favret, Sergei V. Dzyuba

**Affiliations:** Department of Chemistry and Biochemistry, Texas Christian University, Fort Worth, TX 76129, USA; jeanne.m.favret@tcu.edu

**Keywords:** boron-dipyrromethene, fluorescence, imaging, sensors, squarylium dyes, water-soluble dyes

## Abstract

Phosphorus-containing fluorophores provide a versatile framework for tailoring photophysical properties, enabling the design of advanced fluorogenic materials for various applications. Boron dipyrromethene (BODIPY) and squaraine dyes are of interest due to their multifaceted modularity and synthetic accessibility. Incorporating phosphorus-based functional groups into BODIPY or squaraine scaffolds has been achieved through a plethora of synthetic methods, including post-dye assembly functionalization. These modifications often influence key spectroscopic properties and molecular functionality by expanding their utility in bioimaging, sensing, photosensitization, and theranostic applications. By leveraging the tunable nature of phosphorus-containing moieties, these dyes hold immense promise for addressing current challenges in spectroscopy, imaging, and material designs while unlocking new opportunities for advanced functional systems in chemistry, biology, and medicine.

## 1. Introduction

Organophosphorus compounds are versatile molecules with diverse applications spanning modern sciences, technology, medicine, and everyday life [1,2,3,4,5,6,7,8,9,10,11,12]. Many phosphorus-containing functional groups, along with developed chemistries for their installations and functionalization, provide endless possibilities for phosphorus incorporation into various useful scaffolds and systems. Phosphorus-containing fluorophores have been of interest due to their diverse photophysical properties [13,14,15,16,17].

**Figure 1:**
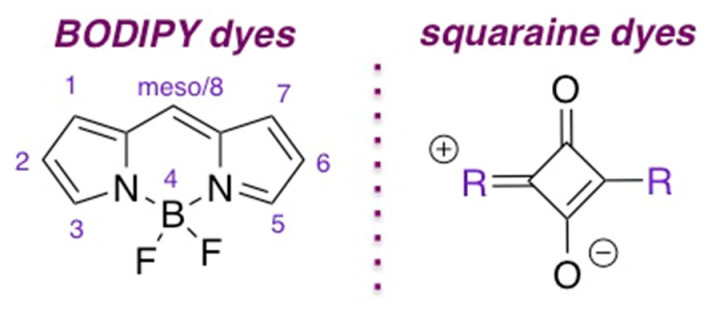
General structures of BODIPY (with the numbering system) and squaraine dyes. R = substituent, functional group.

Among the numerous classes of fluorescent molecules, squaraine and BODIPY dyes (Figure 1) are arguably the most versatile scaffolds due to the tunability of their photophysical properties by introducing structurally and functionally diverse moieties [18,19,20,21,22,23,24,25,26,27]. Interestingly, both dyes were first synthesized by Alfred Treibs and coworkers in 1965 (squaraine) [28] and 1968 (BODIPY) [29].

BODIPY and squaraine dyes possess other notable and important similarities, including relatively straightforward and modular syntheses, as well as possibilities for post-dye assembly modifications [18,19,20,21,22,23,24,25,26,27]. BODIPY dyes provide multiple points for functionalization employing a range of synthetic organic reactions, whereas post-functionalization strategies of squaraine dyes could be relatively restricted due to the electrophilicity of the four-membered central ring. Nonetheless, the incorporation of this versatile fluorogenic scaffold still provides a wide range of opportunities for generating systems of wide utility.

This review summarizes accounts on BODIPY and squaraine dyes that feature various phosphorus-containing moieties. Although the scope of P-containing squaraines is comparably narrower than that of the P-containing BODIPY dyes, the research on squaraine analogues could rapidly expand owing to the straightforward introduction of various functionalities, thereby generating significant contributions within the fields of spectroscopy, imaging, and sensing.

## 2. General Synthetic Routes to BODIPY and Squaraine Dyes

**Scheme 1:**
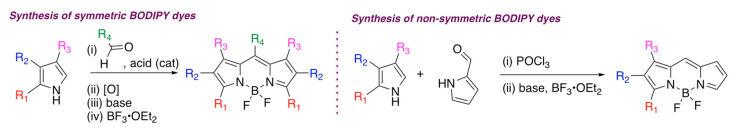
General syntheses of BODIPY dyes. R_1_, R_2_, R_3_, R_4_ = substituent, functional group; [O] = oxidizing agent.

Synthesis of symmetric BODIPY dyes generally relies on an acid-catalyzed condensation reaction between carbonyl-containing compounds (primarily aldehydes and acid chlorides) and various pyrroles, followed by the oxidation that generates a conjugated system and subsequent installation of BF_2_-moeity under basic conditions (Scheme 1, left) [18,19,20,21,22]. Non-symmetric BODIPYs are synthesized in a similar manner (Scheme 1, right). Typically, these syntheses of BODIPYs could be accomplished in one pot, either using solution and solid (mechanochemical) approaches, followed by chromatographic purification.

**Scheme 2:**
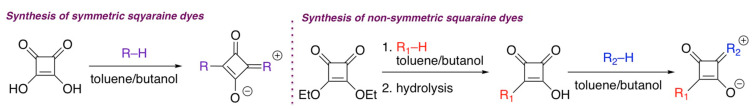
General syntheses of squaraine dyes. R, R_1_, R_2_ = substituent, functional group.

Synthesis of symmetric squaraine dyes is typically accomplished via a one-step condensation of various, typically strong, nucleophiles with squaric acid (Scheme 2, left) [23,24,25,26,27]. Non-symmetric squaraines are synthesized via multistep processes (Scheme 2, right). In general, toluene/n-butanol mixtures are used as solvents (Scheme 2), although other solvents and solvent-free alternatives have been reported. Desired squaraines are isolated either by precipitation, followed by extensive washings with solvents or column chromatography.

## 3. BODIPY and Squaraine Dyes with Phosphorus Bound to Carbon

### 3.1. Phosphonate-Containing Dyes

Neutral (or zwitterionic) dyes, such as BODIPYs and squaraines, usually possess limited solubility in aqueous milieu, which hinders their use in many biological applications. To overcome these limitations, a number of water-soluble versions of such dyes have been reported [30,31,32,33,34,35].

**Scheme 3:**
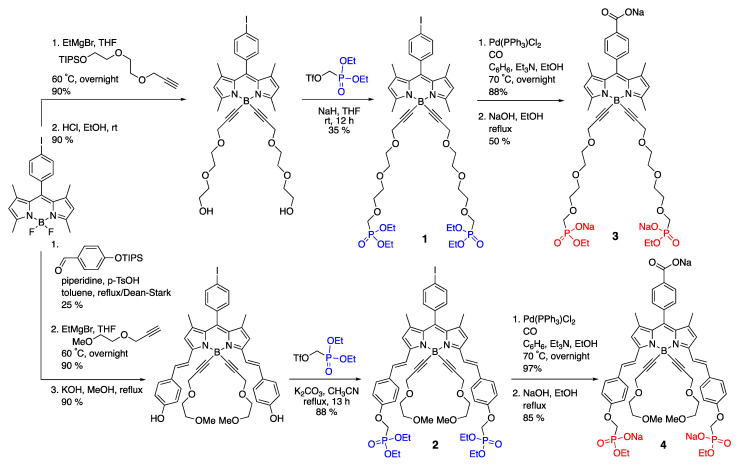
Synthesis of water-soluble, phosphonate-containing BODIPY dyes.

**Scheme 4:**
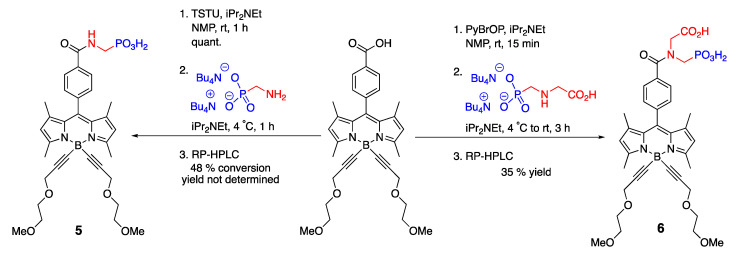
Synthesis of water-soluble BODIPY dyes via incorporation of phosphonate group at the *meso*-substituent. TSTU: O-(N-Suc-cinimidyl)-1,1,3,3-teramethyl-uronium tetrafluoroborate, NMP: N-methyl-2-pyrrolidone, PyBrOP: (benzotriazol-1-yloxy)tripyrrolidinophosphonium hexafluorophosphate.

Incorporation of phosphonate groups onto the dyes’ scaffolds resulted in water-soluble BODIPY dyes **1**–**4** [36] (Scheme 3) as well as **5** and **6** [37] (Scheme 4), with the phosphonate groups being incorporated into various positions of the BODIPY scaffold. Not surprisingly, λ_abs_^max^/λ_em_^max^ for dyes **3** (500 nm/509 nm) and **4** (638 nm/654 nm) appeared to be very similar to typical BODIPY and styryl-BODIPY dye due to the remote, non-conjugated to BODIPY-scaffold nature of the phosphonate group. Due to the high polarity of the phosphonate group, drawbacks were encountered that potentially limit the utilization of these types of dyes. Specifically, the high polarity of the phosphonate group, while enhancing water solubility, often compromises dye stability under physiological or acidic conditions and limits membrane permeability, posing significant challenges for both synthesis and biological applications. Dye **5** was found to be unstable, resulting in a yield that was too low to quantify following HPLC purification [37]. Dye **6** appeared to be more stable, yet reverse-phase chromatography had to be used to obtain the pure dye.

Most photophysical properties, such as λ_abs_, λ_em_, fluorescence lifetime (τ), and fluorescence quantum yield (Φ_f_) of **3** and **4** [36] in H_2_O, were found to be virtually identical to those found in MeOH or EtOH (solvents in which BODIPY dyes are typically very soluble), suggesting the aggregation state of these dyes was similar to that found in polar organic solvents.

On the other hand, dye **6** (Scheme 4) exhibited a superior quantum yield (i.e., Φ_f_ = 0.76) compared to other prepared water-soluble BODIPYs (Φ_f_ was in the 0.36–0.59 range) [37]. This was attributed to a restricted rotation of the tertiary amide moiety, which was theorized to have reduced the probability of non-radiative pathways. Despite the dye’s advantageous level of brightness, it was mentioned that the phosphonic acid group could be an impediment to cell permeability of this dye, potentially limiting its used as a probe in imaging applications.

**Scheme 5:**
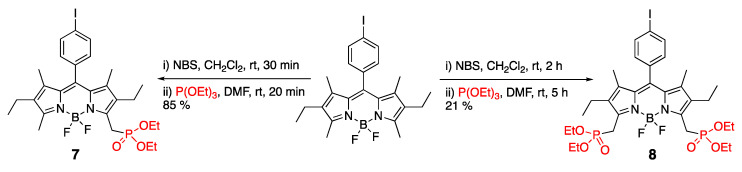
Synthesis of phosphonate-containing BODIPYs via functionalization of 3-/5-methyl substituents. NBS: N-bromosuccinimide.

The varying reactivity of the methyl groups at different positions on the BODIPY scaffold enabled selective bromination at the 3- and 5-positions, followed by reaction with triethyl phosphite to yield mono- and di-phosphonate-containing BODIPY dyes **7** and **8** (Scheme 5) [38].

**Scheme 6:**
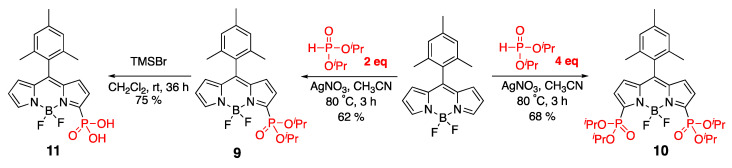
Direct phosphorylation of BODIPY scaffold.

Silver-mediated one-step phosphonate-group installation onto the BODIPY scaffold provided entry to mono- and di-substituted (i.e., **9** and **10**, respectively) dyes by simply adjusting the amounts of dialkyl phosphite (Scheme 6) [39]. Furthermore, **9** was converted into a water-soluble **11** (Scheme 6), and the high solubility of **11** in aqueous media (water, pH 6 and pH 8) was supported by the fact that the photophysical properties of **11** in water were closely related to those observed in DMSO.

**Scheme 7:**
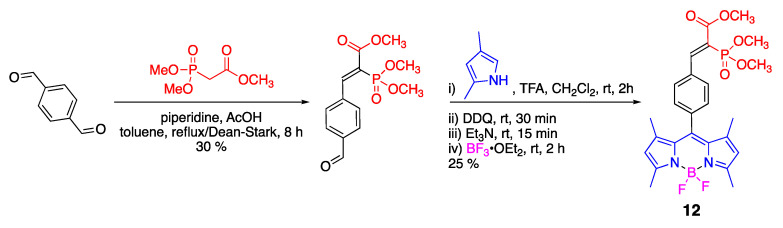
Synthesis of BODIPY **12** using phopshonate-containing aldehyde. TFA: trifluoroacetic acid, DDQ: 2,3-dichloro-5,6-dicyano-p-benzoquinone.

Unlike the aforementioned examples, which relied on modification of the BODIPY scaffolds, an alternative, more modular strategy utilized the incorporation of phosphonate group onto terephthalaldehyde, which was subsequently used to make BODIPY dye **12** (Scheme 7) [40]. λ_abs_^max^/λ_em_^max^ **12** (488–492 nm/511–518 nm) in several solvents (e.g., toluene, CH_3_CN, EtOH, and CH_2_Cl_2_) indicated that the phosphonate group did not have an appreciable impact on its photophysical properties.

**Figure 2:**
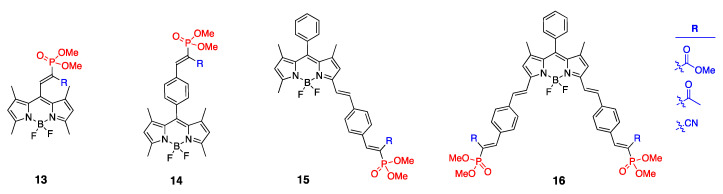
Structures of phosphonate-containing BODIPY dyes utilized in computational studies.

Several phosphonate-containing BODIPY dyes, i.e., **13**–**16**, were investigated computationally using DFT and DFT-TD calculations (Figure 2) [41]. Comparable photophysical properties to experimental data were obtained using HSEH1PBE functional in combination with 6-31+G** basis set. However, the specific effects of the phosphonate groups might be hard to decouple from other functional groups.

**Scheme 8:**
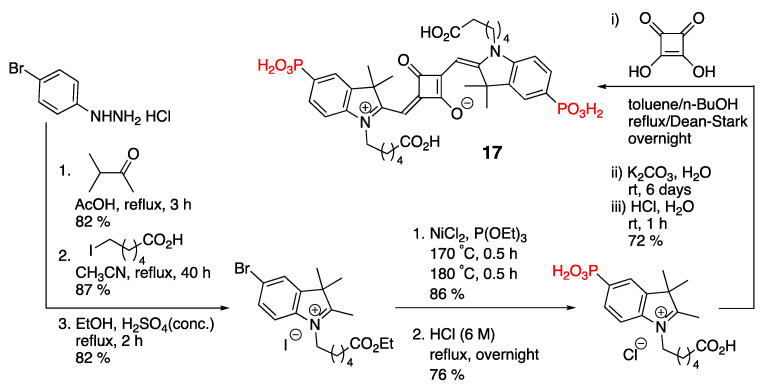
Synthesis of water-soluble, phosphonate-containing squaraine dye **17**.

Examples of phosphonate-containing squaraines have been limited. Synthesis of **17** was accomplished using standard protocols for indolinium types of squaraine dyes (Scheme 8) [42]. Dye **17** was found to be soluble in aqueous media within the 4.0–9.3 pH range and yet insoluble in the media with pH 1.0 (0.1 M HCl). Both absorption and emission maxima appeared to be pH-independent (λ_abs_^max^/λ_em_^max^: 637/647 nm at pH 9.3 (0.1 M Na_2_CO_3_/0.1 M NaHCO_3_ buffer), 635/644 nm at pH 7.3 (PBS buffer), and 634/643 nm at pH 4.0 (1 M NaH_2_PO_4_)). Fluorescence quantum yield for **17** was found to be relatively low (Φ_f_ = 0.04) over the same 4.0–9.3 pH range. However, when **17** was conjugated to sheep γ globulin (S-IgG) in a 1:1 ratio (conversion of **17** to NHS-**17** ester was accomplished using standard coupling conditions: TSTU (O-(N-succinimidyl)-1,1,3,3-teramethyluronium tetrafluoroborate), iPr_2_NEt, DMF/H_2_O, to give mono/di-NHS ester in ca. ¼ ratio) the quantum yield of the obtained conjugate (**17**–S-IgG), increased almost four-fold (Φ_f_ ca. 0.16) in both pH 7.3 and 9.3 buffers. However, a gradual decrease of the quantum yield to original value of 0.04 was observed with the increase **17**/S-IgG ratio (from 1:1 to 5:1), suggesting that aggregation could have been responsible for the low quantum yield of **17**.

### 3.2. Phosphine-Containing BODIPY Dyes

**Scheme 9:**
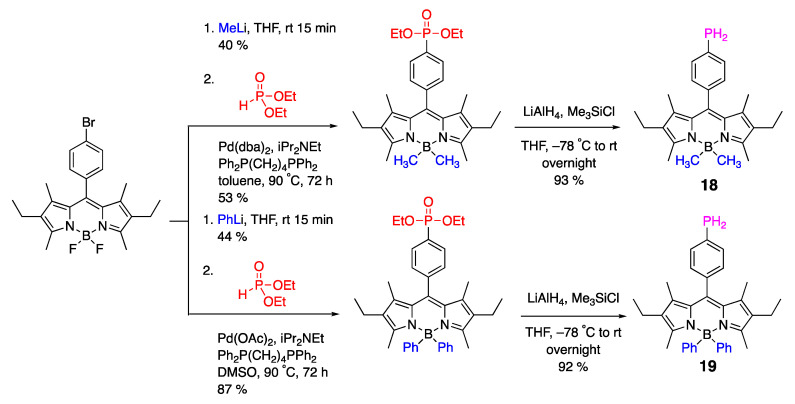
Synthesis of air-stable primary phosphine-containing BODIPYs **18** and **19**.

Although primary phosphines are known for their high reactivity and low air stability, dyes **18** and **19** demonstrated remarkable air stability in both solution and solid states, with no oxidation taking place over a week as well as during purification using column chromatography (Scheme 9) [43]. Additionally, DFT calculations performed on **18** and **19** revealed that phosphorus contribution was prominent only in HOMO-3 (**18**) and HOMO-7 (**19**), consistent with a reduced oxidation propensity of the P-center [44].

**Scheme 10:**
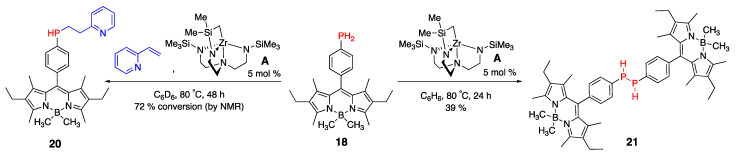
Zirconium-catalyzed hydrophosphination and dimerization of **18**.

**Figure 3:**
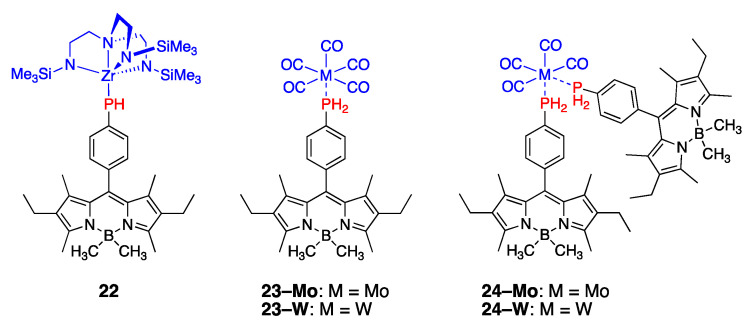
Structures of Zr-, Mo-, and W-containing BODIPY dyes.

Dye **18** was also shown to undergo several additional transformations enabled by zirconium catalyst **A** (Scheme 10) [45]. **A** catalyzed hydrophosphination and dimerization of **18** to give dyes **20** and **21**, respectively [45]. λ_em_^max^ for these dyes (i.e., **20** and **21**) were very similar to the parent dye **18**. ^31^P and ^1^H NMR study (as well as LC-MS) indicated that **21** existed as a mixture of *rac* and *meso* isomers. Thus, the potential rotor-like behavior, along with their associated applications as viscosity and temperature sensing, could be of interest. The zirconium-containing dye **22** (Figure 3), suggested to be the reactive catalytic species, was synthesized by simply reacting equimolar amounts of **18** with **A** (Scheme 10) in toluene for 10 min, following low-temperature crystallization. However, no photophysical properties for **22** were reported.

The ability of **18** to form air-stable fluorescent complexes with other metals was also demonstrated (Figure 3) [46]. Molybdenum- and wolframium-containing mono- and di-BODIPY complexes **23** and **24** were prepared in a facile manner, using photochemical and thermal approaches, respectively, and then isolated using column chromatography. The photophysical properties of these dyes were nearly identical to **18**, indicating the lack of interaction between metal–phosphine moieties and BODIPY core.

**Scheme 11:**
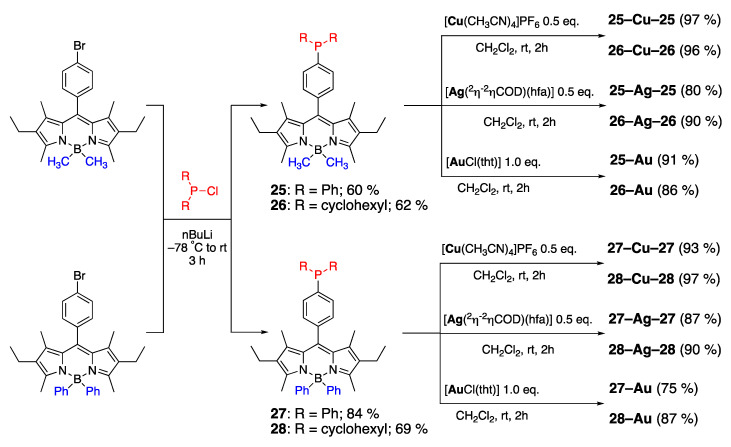
Synthesis of tertiary phosphine-containing BODIPY dyes **25**–**28** and their respective complexes with coinage metals; the isolated yields of the complexes are given in parentheses. COD: cyclooctadiene, hfa: hexafluoroacetylacetonato, tht: tetrahydrothiophene.

Tertiary phosphine-containing BODIPY dyes **25**–**28** were investigated for their ability to form various complexes group 11 metals, i.e., Cu, Ag, and Au, also known as coinage metals (Scheme 11) [47]. Monomeric and dimeric metal complexes were obtained by simply controlling the stoichiometries. Notably, isolation and purification of these complexes was performed either by recrystallization or column chromatography. In general, it was shown that the incorporation of the metal center had a negligible effect on the λ_abs_^max^/λ_em_^max^ and quantum yields of these dyes, yet the extinction coefficients for dimeric complexes were nearly doubled compared to the parent dyes.

**Scheme 12:**
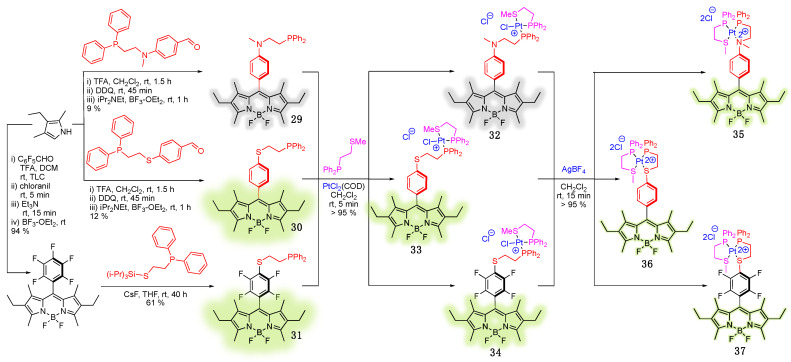
Synthesis of phosphino-amine/thioether-containing BODIPY dyes **29**–**31** and their Pt-complexes. Green glow indicates high-emissive (Φ_f_ > 0.10) dyes; grey glow indicates low-emissive (Φ_f_ < 0.07) dyes; the extent of glow is directly proportional to Φ_f_. TFA: trifluoroacetic acid, DDQ: 2,3-dichloro-5,6-dicyano-p-benzoquinone, COD: cyclooctadiene.

Tethering phosphine moieties to the BODIPY scaffold [48] enabled the investigation of how metal complexation could be used to control fluorescence emission (Scheme 12). Phosphino-amine **29** and phosphino-thioethers **30** and **31** both demonstrated complimentary OFF-ON fluorescence responses upon specific complexation with Pt(II), with these responses being further modulated by chloride ions through induced coordination changes. Specifically, emission (fluorescence quantum yield, Φ_f_) of **29** was relatively low (Φ_f_ = 0.07), and it was further decreased upon Pt-coordination, i.e., **32** (Φ_f_ = 0.03); yet removal of Cl^–^ from the coordination sphere of Pt resulted in a relatively emissive **35** (Φ_f_ = 0.32). The opposite trend was observed for **30** and **31**: the emission gradually decreased from **30/31** (Φ_f_ 0.83/0.91) to **33/34** (Φ_f_ 0.64/0.76) to **36/37** (Φ_f_ 0.15/0.16) [48].

**Scheme 13:**
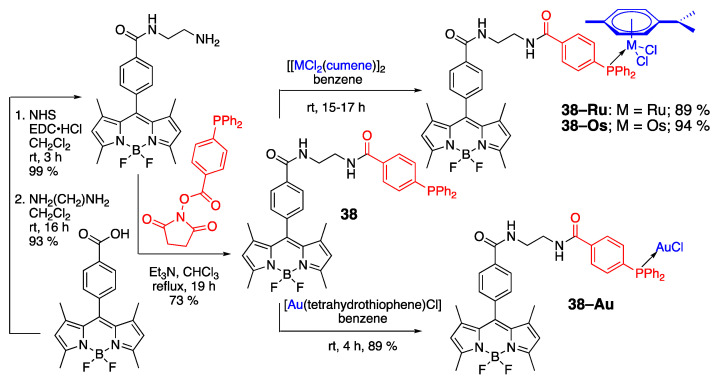
Synthesis of dye **38** and its respective metal complexes. NHS: N-hydroxysuccinimide, EDC: 1-ethyl-3-(3-dimethylaminopropyl)carbodiimide.

In an effort to develop novel metal-containing therapeutic agents, **38** scaffold was prepared [49], followed by incorporation of Ru, Os, and Au to give dyes **38-Ru**, **38-Os**, and **38-Au**, respectively (Scheme 13). As expected, due to the remote position of the phosphine and metal moieties from the BODIPY core, both λ_abs_^max^ and λ_em_^max^ remained relatively unchanged upon metal complexation. Small changes in the quantum yields were attributed to possible photoinduced electron transfer (PET) processes associated with the presence of Ru(II) and Os(II) centers. Confocal microscopy studies revealed that these dyes preferentially accumulate in the cell membrane. Importantly, all three dyes exhibited cytotoxicity toward ovarian cancer cell lines (e.g., A2780S and A2780cisR), albeit the cytotoxicity was inferior to that exhibited by *cis*platin.

**Scheme 14:**
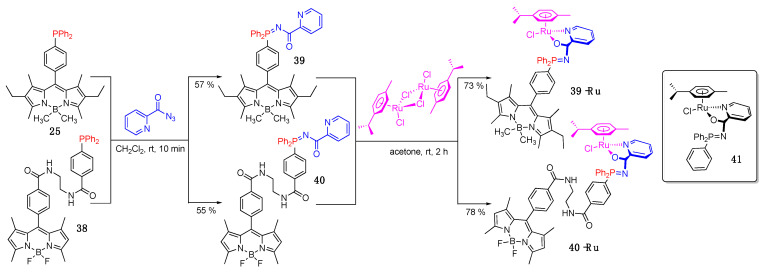
Synthesis of **39-Ru** and **40-Ru** BODIPY dyes and the structure of non-fluorescent Ru-drug candidate **41**.

To aid in the visualization of Ru-containing drug candidates, several BODIPY dyes were prepared (Scheme 14) and evaluated for their toxicity toward cancer lines as well as their ability to suppress cancerous tumor growth [50]. Specifically, dyes **25** and **38** underwent Staudinger reaction with 2-pyridinecarbonyl azide to give dyes **39** and **40**, which were subsequently metalated, to produce air-stable **39-Ru** and **40-Ru**, respectively (Scheme 14) [50]. Although λ_abs_^max^/λ_em_^max^ did not change appreciably as the PPh_2_ functionality was gradually elaborated into iminophosphorane and Ru-iminophosphorane, the quantum yield decreased appreciably upon incorporation of the Ru center. The quenching effect is especially pronounced for **39-Ru**, where Ru is located nearer to the BODIPY core (i.e., direct interaction between the metal center and the BODIPY), which was attributed to PET and/or increased contribution of the triplet states. Confocal microscopy revealed that, similarly, these dyes accumulated in the nucleus, mitochondria, and cytoplasm. Although BODIPY allowed for fluorescent tagging of the Ru-complexes, BODIPY moiety had a negative impact on cytotoxicity (e.g., IC_50_ values for **39-Ru** and **40-Ru** were over an order of magnitude higher as compared to **41** in several cell lines, including triple-negative breast cancer/MDA-MB-231, non-tumorigenic breast/MCF10a, and lung fibroblasts/IMR-90).

**Scheme 15:**
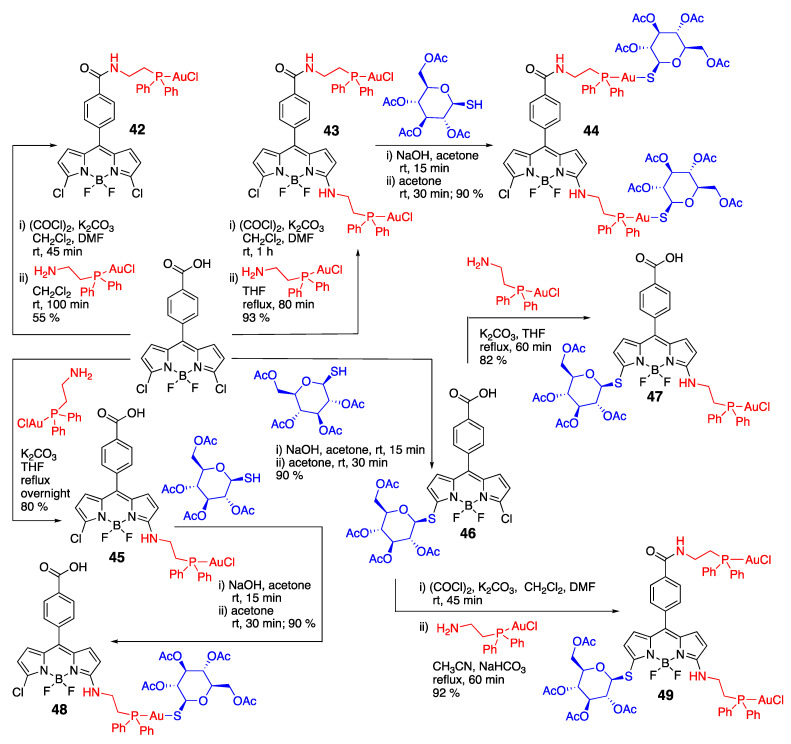
Synthesis of multifunctional Au-containing BODIPY scaffolds.

Leveraging multiple functionalization points of the BODIPY core, more advanced theranostic BODIPY platforms were synthesized, featuring tunable selectivity, solubility, cytotoxicity, and optical imaging capabilities (Scheme 15) [51]. The photophysical properties of **42**–**49** (λ_abs_^max^/λ_em_^max^ were in the range of 475–516 nm/533–570 nm) did not show significant correlations as a function of structural variation, and they were acceptable for in vitro investigations. Confocal imaging revealed that these dyes mostly accumulated in the cytoplasm and/or membrane. Notably, the presence of two Au centers, i.e., **43**, **44**, and **49**, did not provide increased Au uptake. Although Au uptake was found to correlate with cytotoxicity, the cytotoxicity of these dyes toward human breast, murine mammary, and human melanoma cancer cell lines was relatively low, with IC_50_ values being in the 20–100 μM range, indicating limited cytotoxic potential [51].

**Scheme 16:**
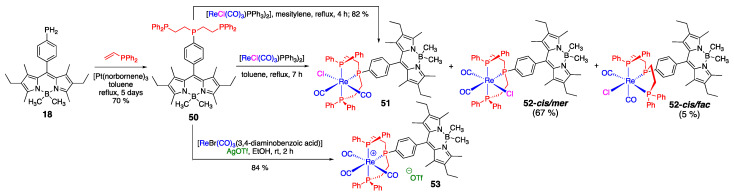
Synthesis of re-containing isomeric dyes **51** and **52** (*cis/mer* and *cis/fac* isomers) and **53**. The yield of **51** in toluene synthesis was not provided; the isolated yields for **52** isomers are given in parentheses.

Due to the potential of rhenium complexes being used as theranostic agents [52], primary phosphane-containing BODIPY **18** was converted into triphosphine ligand **50**, facilitating the synthesis of **51** (Scheme 16) [43,53]. From the synthetic standpoint, it is notable that simply switching the solvent (from mesitylene to toluene) allowed to obtain isomeric dyes **52-*cis,mer*** and **52-*cis,fac*** (along with **51**), which were separated using column chromatography [53]. A related dye **53** was obtained under similar conditions (Scheme 16) [53]. Photophysical properties of all dyes, i.e., **51**–**53**, were nearly identical (λ_abs_^max^/λ_em_^max^: 512–513 nm/526–528 nm; Φ_f_: 0.18–0.28), and suitable for bioimaging applications. Specifically, **51** and **53** were shown to be viable imaging agents for prostate carcinoma (PC-3) cells. However, it appeared that although **51** possessed no cytotoxicity toward PC-3 cells, **53** was found to cause some morphological changes in PC-3 cells and exhibited MI_50_ values (concentration that reduces mitochondrial metabolism to 50%) of 45 ± 5 μM.

**Scheme 17:**
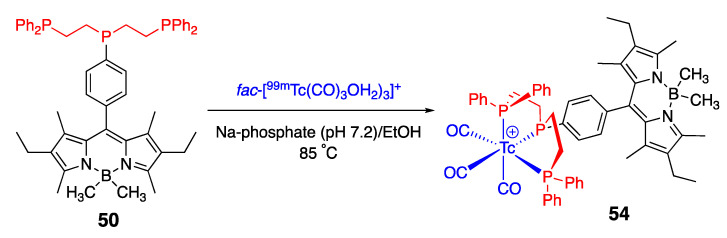
Synthesis of ^99m^Tc-containing BODIPY dye **54**.

In light of utilizing ^99m^Tc-containing ligands in single-photon emission computed tomography (SPECT), dye **54** was prepared (Scheme 17) [53]. Radio-HPLC, under in vitro simulated conditions (1 mM cystine, 10 mM [PO_4_]^3–^, pH 7.2, 37 °C), revealed that **54** remained stable up to 18 h. Thus, **54** was proven to be a viable bimodal (fluorescence imaging/BODIPY and Tc-metal/SPECT) biological probe.

**Scheme 18:**
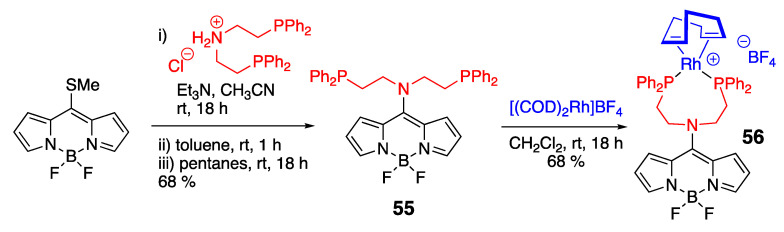
Synthesis of BODIPY dyes **55** and **56**.

A structurally similar amino-diphosphine BODIPY **55** was used for the synthesis of Rh-containing **56** (Scheme 18) to explore the potential of the BODIPY scaffold in controlling catalytic activity [54]. Notably, the fluorescence quantum yield of **56** (Φ_f_ = 0.07) was higher than that of **55** (Φ_f_ = 0.04), although both exhibited low quantum yields in absolute terms. This was attributed to the flexibility of the nitrogen substituents, which likely facilitated non-radiative decay and limited fluorescence efficiency. Upon complexation with Rh, the reduced flexibility of the side arms in **56** appeared to mitigate that effect. Importantly, **56** demonstrated efficient catalytic activity in the hydroboration of alkenes (e.g., styrene, 1-hexene, (+)-limonene, and β-pinene), albeit its performance was inferior in hydrogenation, hydrosilylation, and hydroacylation reactions [54].

**Figure 4:**
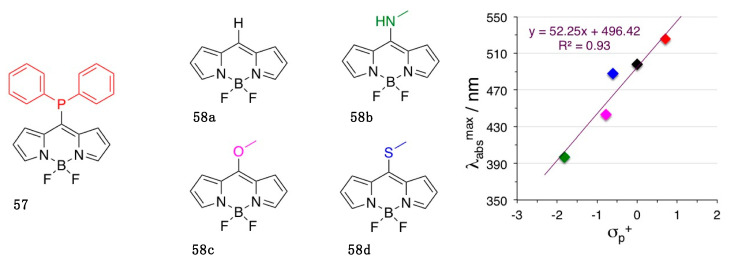
Structures of dyes **57**, **58**, and the Hammett plot relating electronic effects of the *meso*-substituents and λ_abs_^max^. Colors of the symbols in the graph correlate with the color of the *meso*-substituent in the structures.

Dye **57**, where P of the phosphine group is directly attached to the BODIPY core, was investigated along with other dyes **58a–d** that varied by the heteroatom (i.e., N, O, and S) in the *meso*-position of the BODIPY core (Figure 4) [55].

Compared to the parent **58a**, λ_abs_^max^ of **57** was red-shifted compared, while dyes with other heteroatoms, i.e., **58b–d**, exhibited a pronounced blue shift. These spectral differences correlated with the electron-donating or electron-withdrawing nature of the substituents in the *meso*-position of the BODIPY core, as evidenced by a strong correlation with the Hammett σ_p_^+^ parameter (Figure 4). These trends were further supported by cyclic voltammetry measurements and quantum mechanical calculations [55].

In sharp contrast to **58a–d**, dye **57** appeared to be non-fluorescent, evident by a drastic decrease of the quantum yield (i.e., ca. 300–900-fold decrease compared to **58a**). Despite this, the reported λ_em_^max^ values suggested a red shift for **57** (i.e., 559 nm vs. 508 nm), consistent with the electronic effects of *meso*-position substituents on the BODIPY core. The non-emissive nature of **57** was attributed to the internal rotation of phenyl groups, which induced non-radiative deactivation mechanisms [55].

**Scheme 19:**
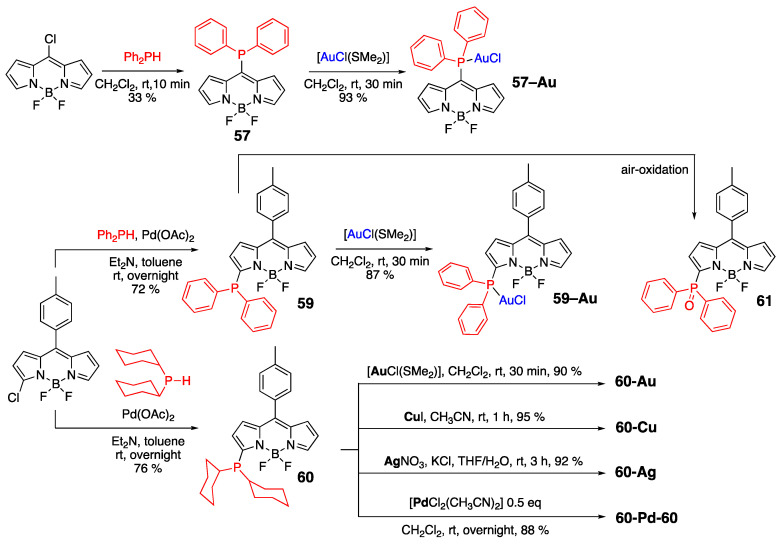
Synthesis of dyes **58**, **59**, and **60** and their respective metal complexes. **61** was obtained as a side product from reactions involving **59** as the starting material.

It was further proposed that the involvement of the non-bonding electrons of phosphorus in PET processes in the excited state could account for the non-emissive nature of dye **57** and related dyes **59** and **60** (Scheme 19) [56]. Electronic communication between phosphorus and the BODIPY core was supported by ^31^P NMR, which showed a triplet associated with a long-range P–F coupling (e.g., **59** ^4^J_P–F_ = 45 Hz and **60** ^4^J_P–F_ = 52 Hz). Notably, this coupling decreased or almost disappeared upon metal complexation with gold (**59-Au** ^4^J_P–F_ = 15 Hz and **60-Au** ^4^J_P–F_ < 2 Hz), and the corresponding dyes exhibited pronounced enhancement of the fluorescence emission. Interestingly, the nature of the metal appeared to strongly contribute to emission of **59-metal** and **60-metal** complexes. For instance, based on the quantum yields, **60-Au** (Φ_f_ = 0.09) and **60-Ag** (Φ_f_ = 0.07) dyes were more emissive than **60-Cu** (Φ_f_ = 0.02), while dimeric **60-Pd-60** (Φ_f_ < 0.001) was non-emissive. Consistent with these findings, the phosphine oxide derivative of **59**, i.e., dye **61**, where the electron pair on the phosphorus atom was removed from the conjugation with BODIPY, was also fluorescent [56].

**Scheme 20:**
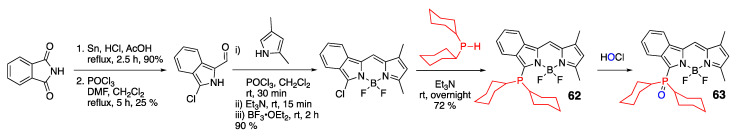
Synthesis of the OFF-ON probe **62** (OFF-state) and its oxidation to **63** (ON-state).

**Scheme 21:**
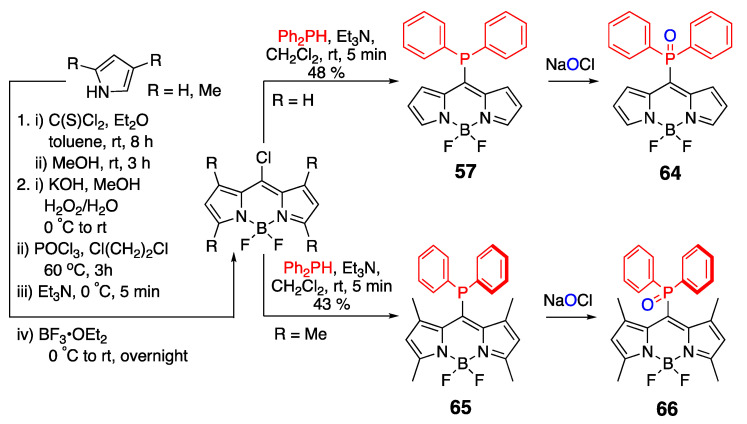
Synthesis of OFF-ON probes **57** (OFF-state) and **65** (OFF-state) and their oxidation to **64** (ON-state) and **66** (OFF-state).

Thus, the oxidation of phosphines to phosphine oxides (e.g., **59** to **61**, Scheme 19) fundamentally represents a viable strategy for designing oxidation-sensitive OFF-ON probes (Scheme 20 and Scheme 21) [57,58]. Incorporating the phosphine group in the 3-position on the BODIPY core (i.e., **62**, Scheme 20) [57] or *meso*-position (i.e., **57** or **65**, Scheme 21) [58] provided OFF-ON probes capable of detecting ClO^–^ species (e.g., NaOCl and HOCl) in both in vitro and in vivo settings via oxidation to the respective P-oxides **63**, **64**, and **66**. Similar to **59** and **60** (Scheme 19, [56]), the coupling between P and F was observed by ^31^P NMR in the case of **62**, which disappeared upon conversion to **63**. From the synthetic perspective, it is noteworthy that the installation of dicyclohexyl phosphine functionality within the synthesis of **62** [57] was achieved without the use of Pd, unlike **60** [56], which required a Pd catalyst.

Furthermore, while **57** exhibited OFF-ON fluorescence response upon oxidation to **64** (i.e., sensing of NaOCl) [58], which is consistent with the removal of PET, its non-rotating analogue **65** (featuring methyl groups in 1- and 7-positions) did not display a fluorogenic response upon oxidation to **66**. This lack of fluorogenic response was attributed to the formation of a distorted structure, which was prone to thermal deactivation processes. However, the absorption of **65** (red solution) underwent a significant red shift (ca. 50 nm) upon conversion to phosphine oxide **66** (blue solution) as a result of NaOCl-induced oxidation. This pronounced shift might suggest the potential utility of **65** as a naked eye-detection sensor [58].

### 3.3. Phosphine Reactions with Squaraine: En Route to Squaraine-Chemodosimeters

**Scheme 22:**
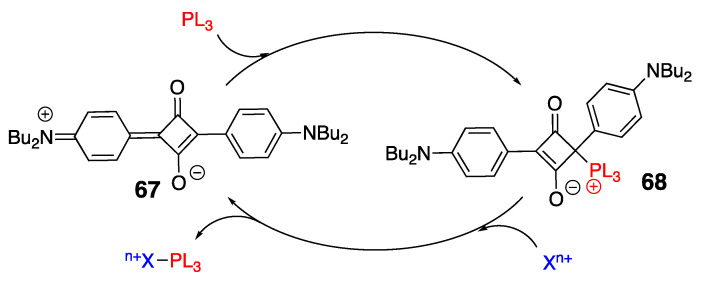
Nucleophilic phosphine addition to squaraine **66**, making metal sensor **67**. L = *^n^*Bu, NMe_2_, *p*-MeOHC_6_H_4_; X: metal ion. See text for details.

The electrophilicity of the four-membered ring in squaraine dyes presents not only challenges for squaraine modification, but it also provides opportunities to expand their structural diversity and applications. For instance, several sensors and chemodosimeters (i.e., chemosensors in which optical responses are induced by the formation of a covalent bond with the analytes) have been developed based on reversible nucleophilic addition of thiols to squaraines. P-containing nucleophiles, such as P(n-Bu)_3_ and P(NMe_2_)_3_, were shown to react with squaraine dyes, such as **67**, to produce novel chemodosimeters **68** (Scheme 22) [59]. Specifically, the rapid and efficient addition of phosphines to squaraine dyes led to stable adducts that were easily isolated simply by solvent removal and subsequent recrystallization. Notably, P(p-MeO-C_6_H_4_)_3_ exhibited a reversible, temperature-dependent, yet less efficient, addition to **67**.

The sensing behavior of **68** (L = *^n^*Bu) was evaluated with a set of transition metal ions [59]. Specifically, Pd^2+^, Rh^+^, Rh^2+^, Ir^+^, Ag^+^, Au^3+^, Cu^+^, and Cu^2+^ (in low mM-range) induced rapid, albeit partial, regeneration of **67**, as evidenced by the increase of λ_abs_^max^ at 656 nm. Among these, Pd^2+^ was the most efficient and Rh^+^ was the least efficient in facilitating the conversion of **68** to **67**. In contrast, no increase in absorption maximum was noted in the presence of Ni^2+^, Fe^3+^, Ti^3+^, and Ti^4+^.

**Scheme 23:**
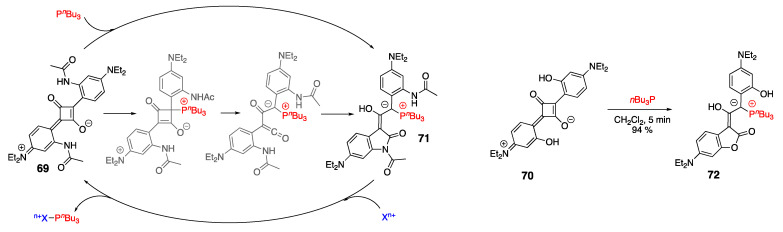
Reaction of squaraine dyes **69** and **70** with the phosphine producing **71** and **72**. Dearomative mechanism (left), based on B3LYP+D3/6-311+G**+CPCM calculation, is shown for conversion of **69** to **71**. X = H, metal. See text for details.

Subsequent studies [60,61] identified that incorporation of *ortho*-NHAc and *ortho*-OH groups onto squaraine scaffold gave dyes **69** and **70** (Scheme 23), which were shown to react with P(*^n^*Bu)_3_ to form **71** and **72** via an unusual dearomative rearrangement [60]. Interestingly, in solution, these P-adducts (e.g., **71**) appeared to exist in equilibrium with the respective squaraines (e.g., **69**), as judged by the concentration-dependent ^1^H NMR spectra. A decrease in concentration of **71** from 100 mM to 100 μM corresponded to a 0–25% increase in **69**. Furthermore, the absorption spectra of **71** (ca. 5 μM range) exhibited a measurable absorption in the 685 nm range, which corresponded to the λ_abs_^max^ of **69**, respectively.

Importantly, chemodosimeter **71** was effective in detecting low μM-concentrations of Pt^2+^, Ag^+^, Pd^2+^, Hg^2+^, Au^+^, and Rh^+^. (A similar response was observed in the presence of H^+^ as well.) Notably, a straightforward modification of the squaraine scaffold (e.g., **67** to **69**) significantly enhanced the chemodosimetric sensitivity from mM-detection range in the case of **68** [59] to μM-detection range in the case of **71** [61].

### 3.4. Phosphonium-Containing BODIPY and Squaraine Dyes

**Figure 5:**
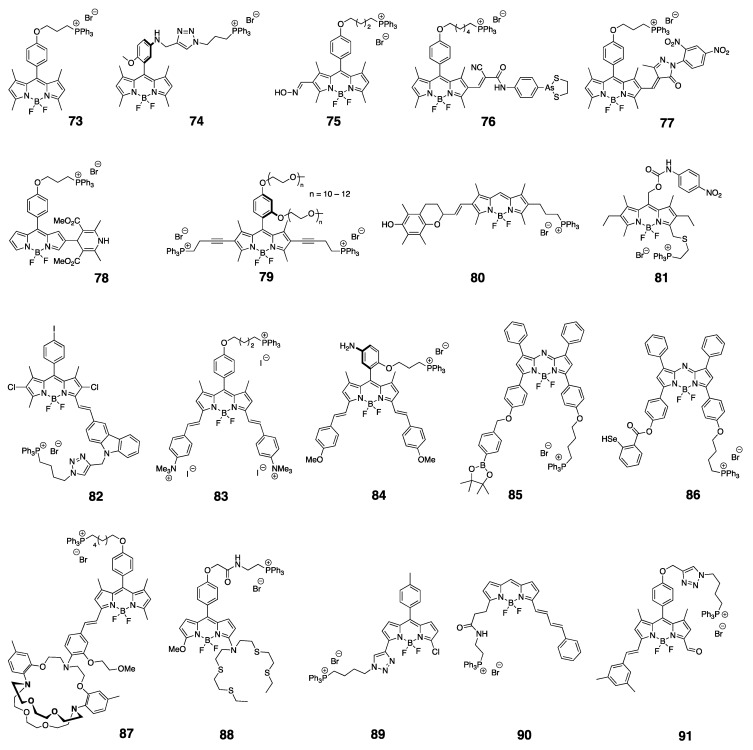
Structures of selected Ph_3_P^+^-containing BODIPY dyes used in mitochondria imaging; citation is given in brackets next to the compound number: **73** [70], **74** [71], **75** [72], **76** [73], **77** [74], **78** [75], **79** [76], **80** [77], **81** [78], **82** [79], **83** [80], **84** [81], **85** [82], **86** [83], **87** [84], **88** [85], **89** [86], **90** [87], **91** [88].

Mitochondria are subcellular organelles critical to a variety of biological functions [62,63,64,65]. Due to its negative membrane potential of mitochondria, incorporation of lipophilic cations onto dyes’ scaffolds allows for specific delivery and imaging of mitochondria. Trisubstituted phosphonium moieties, primarily triphenylphosphonium/(Ph_3_P^+^), have been incorporated onto a wide range of fluorescent dye scaffolds [66,67]. Recently, the use of BODIPY dyes for mitochondrial imaging was comprehensively reviewed [68,69]. Here, selected dyes with distinctly different structural features are highlighted, i.e., **73–91** (Figure 5) [70,71,72,73,74,75,76,77,78,79,80,81,82,83,84,85,86,87,88].

As expected, the incorporation of Ph_3_P^+^ moiety via non-conjugating tethers (typically via ether, amide, or triazole linkages) to the BODIPY core did not alter the photophysical properties of the BODIPY dye scaffold. However, when conjugation was introduced at the 3- and 5-positions, such as styryl-containing dyes **82**–**84**, **87**, and **91** (Figure 5), the emission was shifted toward the near-IR range. Further shifts (i.e., λ_em_^max^ > 700 nm) were achieved by incorporating Ph_3_P^+^ moiety onto aza-BODIPY scaffold, such as **85** and **86** (Figure 5). It is noteworthy that while most BODIPY-Ph_3_P^+^-containing probes exhibited an ON-OFF type of fluorescence responses, some probes, including **89**–**91** (Figure 5), operated via a ratiometric emission mechanism.

**Scheme 24:**
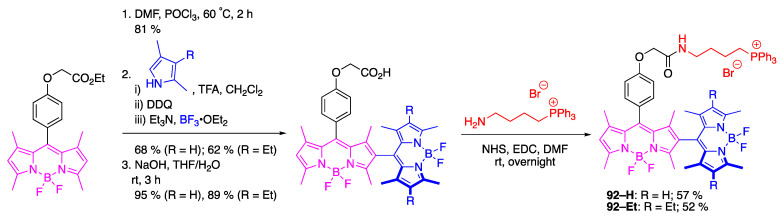
Synthesis of dyes **92-H** and **92-Et**. See text for the NP preparation using these dyes. DDQ: 2,3-dichloro-5,6-dicyano-p-benzoquinone, NHS: N-hydroxysuccinimide, EDC: 1-ethyl-3-(3-dimethylaminopropyl)carbodiimide.

Mitochondrial membrane potential of cancer cells is typically higher than that of normal cells [89]. Consequently, introducing multiple Ph_3_P^+^ groups onto the dyes’ scaffolds may enhance their electrostatic interaction and thus lead to higher localization of these dyes in cancer cells. A recent study [90] examined mitochondria imaging capabilities of nanoparticles (NPs) comprised of dimeric BODIPY dyes **92-H** and **92-Et** (Scheme 24). NPs, with a size of 35–38 nm, were prepared by dissolving the corresponding dyes in acetone, followed by the addition of water and overnight evaporation of acetone. Importantly, zeta-potential studies revealed the Ph_3_P^+^ moieties were positioned at the surface. Due to the twisted structure of the two BODIPY units, the emission spectra of dyes **92-H** and **92-Et** exhibited a significant dependence on the solvent polarity. The emission intensities and quantum yields were drastically lower in polar solvents (e.g., EtOH and CH_3_CN) than in polar solvents (e.g., toluene). However, although the emission intensity of **92-H**-NP and **92-Et**-NP in water was relatively low, especially when compared to the emission of **92-H** and **92-Et** in toluene, it was ca. 3–5-fold higher than the emission intensity of **92-H** and **92-Et** in polar organic solvents (e.g., EtOH and CH_3_CN). Confocal microscopy revealed that both **92** and **92-NP** were localized in mitochondria, proven by co-localization of these dyes with mitochondrial red tracker (MTR) in breast cancer (MCF-7) and cervical cancer (HeLa) cell lines. Additionally, the presence of the twisted di-BODIPY structure, which was advantageous for facilitating the triplet-state formation, thus aided in the efficient generation of ^1^O_2_. As a result, these NP dyes exhibited photoinduced cytotoxicity upon exposure to green LED (λ = 530 nm) light, reducing cell viability in MCF-7, and HeLa cells decreased to ca. 20% in the presence of 0.1 μM of **92-H**-NP and **92-Et**-NP.

**Scheme 25:**
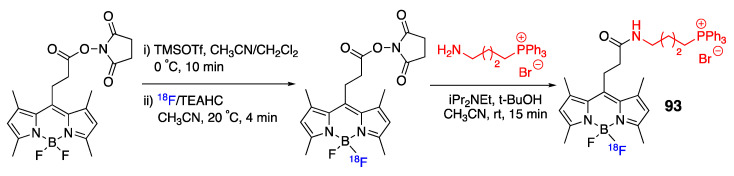
Synthesis of ^18^F-labeld Ph_3_P^+^-containing dye **93**. TEAHC: tetraethylammonium hydrogen carbonate.

In order to aid in the development of multimodal agents, radioactive ^18^F-BODIPY with Ph_3_P^+^ group **93** was prepared (Scheme 25) and successfully evaluated to assess its ability to image myocardial perfusion using positron emission tomography/computed tomography [91].

**Scheme 26:**
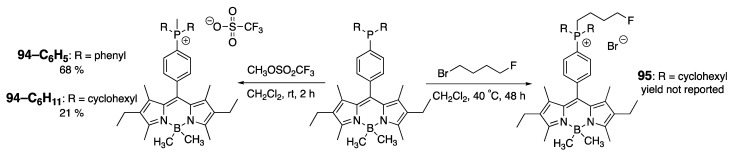
Synthesis of phosphonium-containing dyes **94** and **95**.

Arguably, incorporation of various groups onto phosphonium moiety itself could further tune permeability, compatibility, and localization of the probe. Using **25** and **26** as the starting materials (for synthesis, see Scheme 11), dyes **94–C_6_H_5_** and **94–C_6_H_11_** were synthesized (Scheme 26) to compare the effects of the aromatic and aliphatic groups on MMP-dependent uptake [92]. Increased lipophilicity of the cyclohexyl group, along with the more localized positive charge on phosphorus relative to the phenyl groups, appeared to increase the MMP-dependent uptake of **94–C_6_H_11_** compared to **94–C_6_H_5_**. Importantly, dye **95**, where the methyl group was a longer alkyl group, demonstrated an even greater increase in uptake. Going further, incorporating an F-group could open possibilities for developing multimodal systems (e.g., ^18^F-version, similar to **93** (Scheme 25), albeit not made, should, in principle, provide a fluorescent/PET agent).

**Scheme 27:**
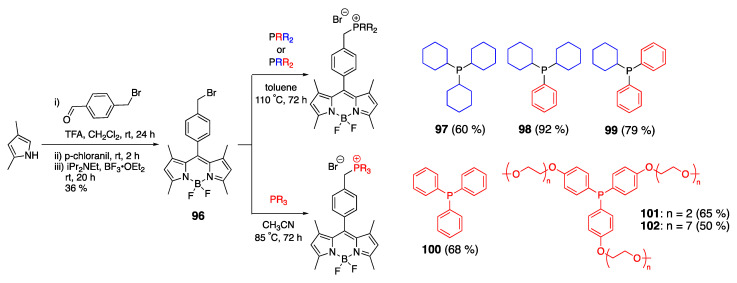
Modular synthetic approach to phosphonium-containing BODIPY imaging probes **97**–**102**. Isolated yields are given in parentheses. TFA: trifluoroacetic acid.

The bromine-containing scaffold **96** was recently prepared following standard BODIPY-type synthetic methods and subsequently functionalized with various phosphonium-containing moieties (Scheme 27) [93]. Since the phosphonium group was not in conjugation with the BODIPY core, λ_abs_^max^/λ_em_^max^ (497–498 nm/511–512 nm) remained virtually unchanged, with a similar trend observed for the quantum yields. However, by tuning the lipophilicity of the groups on the phosphonium moiety, it was possible to establish structural features important for probe localization and mitochondria membrane potential (MMP) sensitivity. Among **97**–**100** probes, **98** displayed the largest sensitivity to the MMP, whereas **102** showed both superior biocompatibility and enhanced membrane potential compared to all other probes [93]. This highlighted the potential importance of ethylene glycol pendants for tuning the properties of BODIPY probes in mitochondrial imaging.

**Scheme 28:**
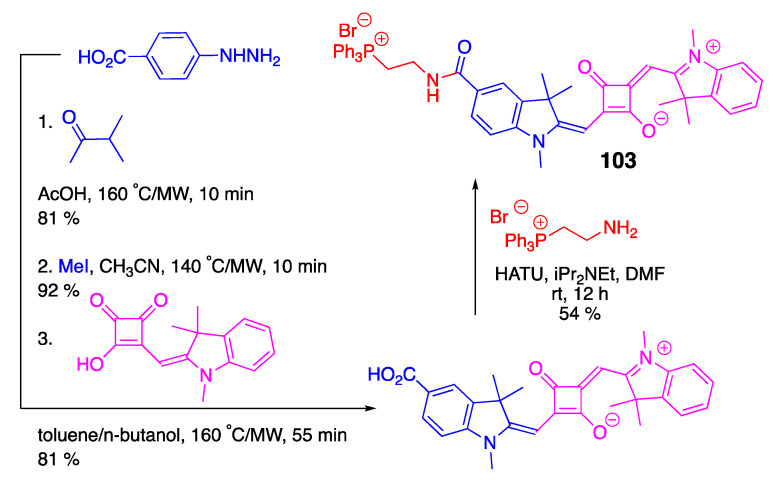
Synthesis of Ph_3_P^+^-containing squaraine **103**. MW: microwave heating, HATU: 1-[bis(dimethylamino)methylene]-1H-1,2,3-triazole[4,5-b]pyridinium 3-oxid hexafluorophosphate.

Relatively fewer examples of squaraine dyes that featured Ph_3_P^+^ moiety have been reported as probes for imaging mitochondria. However, the squaraine scaffold offers unique structural and functional possibilities, such as shifting λ_em_^max^ toward the near-IR window and tuning water solubility. Specifically, non-symmetric squaraine **103** was prepared using conventional approaches to such scaffolds, with the Ph_3_P^+^ group incorporation achieved using standard amide coupling conditions (Scheme 28) [94]. λ_abs_^max^/λ_em_^max^ of ca. 645 nm/655 nm place **103** in between BODIPY and aza-BODIPY mitochondria probes (Figure 5). Although selective localization of **102** in mitochondria was confirmed by confocal imaging, the emission intensity was drastically reduced due to interaction with nucleophilic biothiols.

**Scheme 29:**
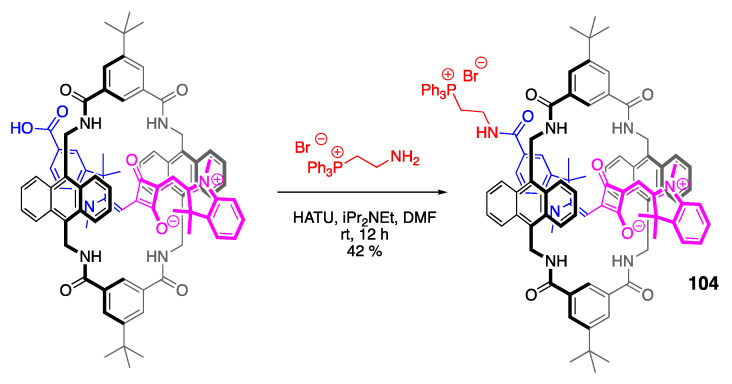
Synthesis of Ph_3_P^+^-containing rotaxane encapsulated squaraine **104**. HATU: 1-[bis(dimethylamino)methylene]-1H-1,2,3-triazole[4,5-b]pyridinium 3-oxid hexafluorophosphate.

Rotaxane encapsulation has been demonstrated to be a viable strategy for preserving the chemical integrity of the squaraine-conjugated structure [95,96,97]. Accordingly, probe **104** was synthesized using solid-state approach (Scheme 29) [94]. As expected, **104** exhibited red-shifted absorption and emission maxima, along with an increased quantum yield, likely a result of restricted rotation of **104** in the interlocked assembly. Notably, no dethreading of **104** from the rotaxane was observed, and the probe exhibited good mitochondria localization in carcinoma HeLa and lung adenocarcinoma (A549) cells.

**Figure 6:**
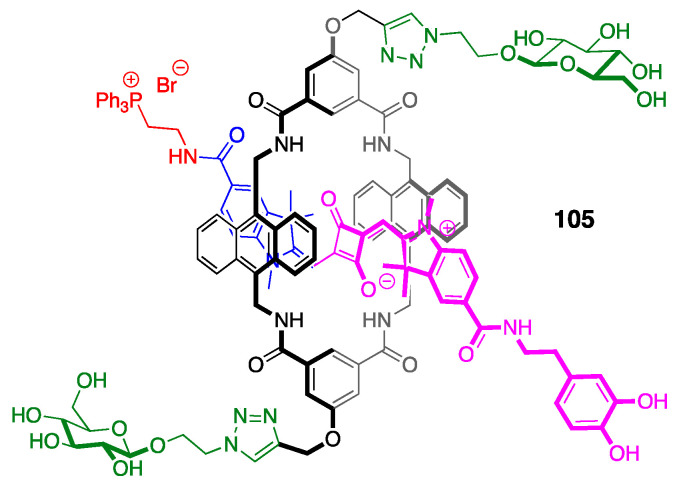
Structure of **105** used for the preparation of multimodal nanoparticles.

Subsequent studies focused on the incorporation of interlocked Ph_3_P^+^-containing squaraine onto superparamagnetic Fe_3_O_4_ nanoparticle to produce **105** for multimodal (i.e., fluorescence emission and T2-weighted magnetic resonance) (Figure 6) [98]. **105** exhibited photophysical characteristics to **104** and selectively stained live-cell mitochondria. As an MRI probe, **105** exhibited higher relaxation times compared to previously used Fe_3_O_4_-nanoparticle agents, such as Feridex and Resovist. Overall, the high water-solubility, good biocompatibility, and multimodal imaging capabilities, combined with the potential heat-generating nanoparticle core, suggested that **105** and related systems could serve as viable theranostic agents.

**Scheme 30:**
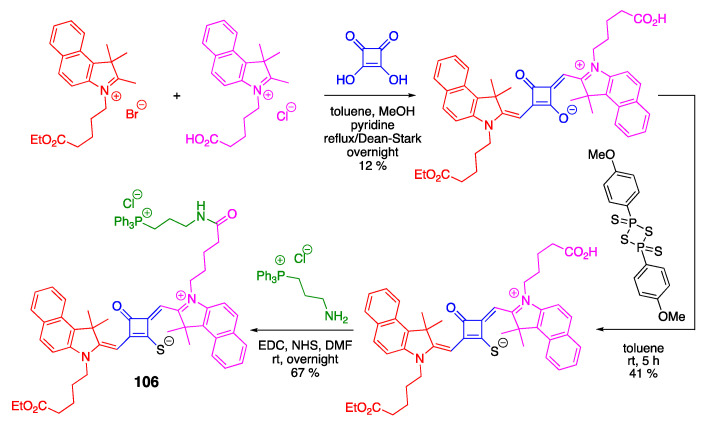
Synthesis of Ph_3_P^+^-containing thiosquaraine **106**. NHS: N-hydroxysuccinimide, EDC: 1-ethyl-3-(3-dimethylaminopropyl)carbodiimide.

In light of the increased photostability of thio-containing squaraines at the single molecule imaging resolution [99], implementing the Ph_3_P^+^-containing squaraine **106** as a mitochondria probe was assessed (Scheme 30). Remarkably, **106** effectively accumulated in HeLa and U2OS cells while simultaneously tracking the mitochondria movement in dorsal root ganglion neurons.

## 4. Fluorescent Dyes with Phosphorus Not Bound Directly to Carbon

### 4.1. Phosphate-Containing Groups

#### 4.1.1. Phosphate-Containing BODIPY: Control of Water Solubility

**Scheme 31:**
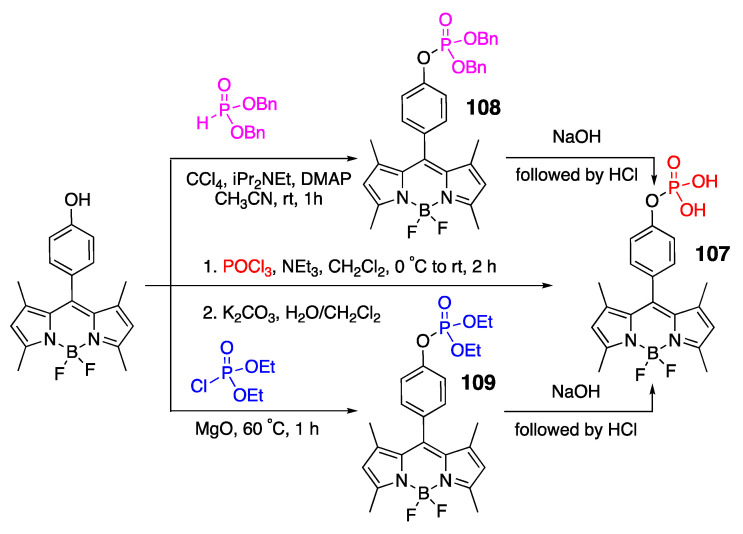
Attempted syntheses of BODIPY **107**.

Several phosphorylation protocols were explored to prepare dye **107** (Scheme 31) [100]. While limited information was provided on the structural characterization of **107** and the intermediates (i.e., **108** and **109**), photophysical studies on **107** revealed that its fluorescence lifetime was ca. five times higher (τ = 3.7 ns) than the lifetime of the corresponding HO-containing BODIPY dye (τ = 0.7 ns) that was used as a starting material in the synthesis of **107** (Scheme 31). This was attributed to the electron-withdrawing nature of PO_3_H_2_-group and, hence, removal of PET. Fluorescence correlation spectroscopy measurements indicated that **107** might be suitable for single-molecule studies. However, the stability of **107** was a significant limitation, as **107** decomposed upon drying.

#### 4.1.2. Phosphate-Containing Dyes: Sensing of Organophosphorus Nerve Agents

**Figure 7:**
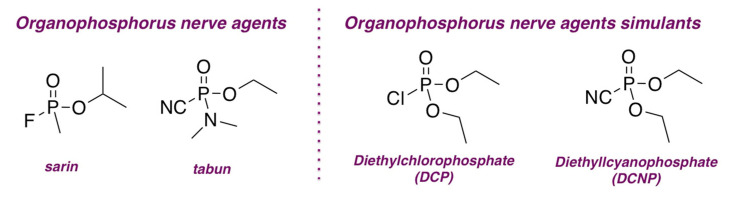
Structures of selected organophosphorus nerve agents and respective simulants.

Phosphorylation of hydroxyl-containing BODIPYs is an important factor to consider as it may alter internal rotations, potentially changing the spectroscopic properties of the dyes. In turn, these changes could open avenues for sensing applications. Specifically, fluorescence-based detection of organophosphorus nerve agents (albeit typically in the form of the nerve agent simulants, Figure 7) using modification of fluorophoric scaffolds has been an area of intense interest [101,102].

**Scheme 32:**
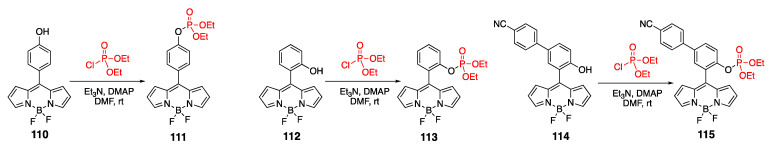
Phosphorylation of HO-containing BODIPY dyes.

The ability of several HO-containing BODIPY dyes, i.e., **110**, **112**, and **113**, to sense nerve agent simulants has been investigated (Scheme 32) [103,104]. The reactivity of the hydroxyl group with the organophosphorus agents was independent of its position on the BODIPY scaffold. However, the mechanisms responsible for the fluorescent responses of these chemodosimeters varied depending on the specific position of the HO group and, as such, on the position of the resulting phosphonate moiety. One such case was dye **112**, which was found to be virtually non-emissive due to the free rotation of the *meso-*substituent around the BODIPY core [103]. Yet, phosphorylation of **112** with diethyl chlorophosphate (Scheme 32) produces dye **113**, in which the size of the phosphonate group significantly restricted that internal rotation. As a result, the non-radiative decays were suppressed, which led to ca. 100-fold increase in fluorescence intensity. The internal rotation within dye **112**, and lack thereof in dye **113**, was further supported by the fluorescence lifetimes in media of varying viscosity. Dye **112** acted as a molecular rotor (and hence a molecular viscometer [105,106]), as evidenced by the linear correlation between the emission intensity and medium viscosity, with a slope of 0.23, consistent with rotor-like behavior [105,106].

In contrast, **113** (i.e., phosphorylated version of **112**) did not exhibit a rotor-like behavior: the emission intensity as a function of the media’s viscosity provided a linear correlation with a slope of 0.026. These findings from steady-state emission measurements were further corroborated by fluorescence lifetime measurements. Notably, both **110** and **111** exhibited rotor-like behavior, with linear coefficients of 0.25 and 0.29, respectively, as a result of viscosity-dependent emission studies. It is significant that the detection of diethyl chlorophosphate vapors was also effectively achieved using paper strips impregnated with **112**: emission was detected within 3–15 sec upon exposure to diethyl chlorophosphate vapors (132 ppm), with a detection limit of ca. 0.25 ppm.

Qualitatively similar results were obtained with a related probe **114** (Scheme 32) [104]. This probe exhibited a slower (540 s) response to DCP (compared to 112, e.g.), yet a very low detection limit (ca. 20 ppb) was reported. Importantly, due to pronounced color changes, the DCP binding could be easily detected by the naked eye using a handheld UV lamp.

**Scheme 33:**
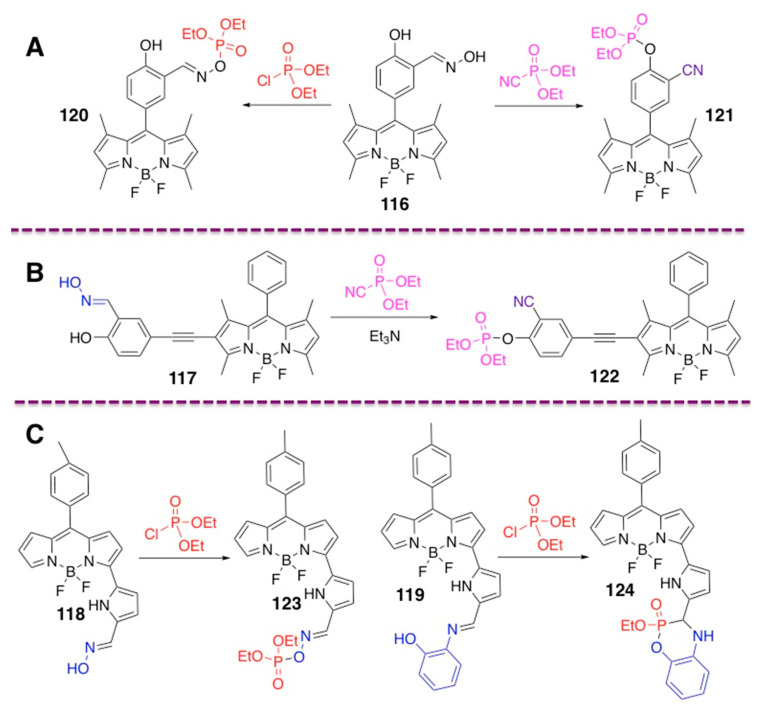
Oxime-containing BODIPY dyes as sensors of DCP and DCNP. (**A**): [107]; (**B**): [108]; (**C**): [109].

Several oxime-containing BODIPY dyes, i.e., **116**, **117**, **118**, and **119**, were evaluated for their ability to sense nerve agent simulants (Scheme 33) [107,108,109]. Due to variations in conditions, direct comparisons of these chemodosimeters may not be feasible. However, reported detection limits for nerve agent simulants’ detection ranged from ca. 1 nM to 90 μM. Interestingly, in the case of **116,** the formed adducts varied depending on the nature of the simulant (i.e., DCP vs. DCNP) [107]. For DCP, phosphorylation of the HO group of the oxime was observed. In contrast, for DCNP, phosphorylation occurred at the phenol group, accompanied by oxidation of the oxime to nitrile. Similar behavior was noted for **117** [108]. In both cases, the electron-withdrawing nature of the nitrile group inhibited the PET process, resulting in the formation of highly emissive species.

**Scheme 34:**
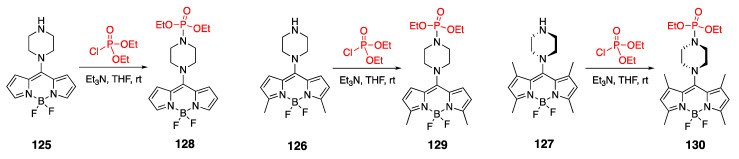
Piperazine-containing BODIPY dyes and their products upon reaction with DCP.

The nucleophilicity of the nitrogen atom in piperazine-substituted BODIPYs was explored in the context of DCP-sensing capability by dyes **125**–**127** to produce the corresponding phosphorylated dyes **128**–**130** (Scheme 34) [110]. Specifically, the internal rotation of the *meso*-substituent was controlled by placing methyl groups in 1- and 7-positions. Since the electron pair on the nitrogen was not in conjugation with the BODIPY core, the protonation was suggested to be a contributing factor to the spectroscopic changes in **127**. Among the investigated compounds, dye **126** provided the highest sensitivity and the most pronounced colorimetric change in response to DCP.

**Scheme 35:**
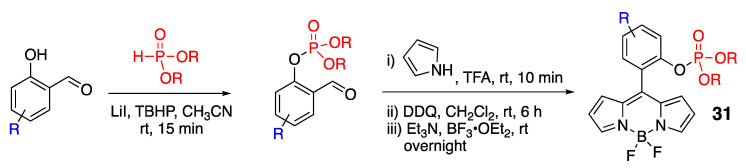
A modular approach to phosphorylated BODIPY dyes with restricted rotations. TBHP: t-butyl hydroperoxide, TFA: trifluoroacetic acid, DDQ: 2,3-dichloro-5,6-dicyano-p-benzoquinone.

The efficient phosphorylation of substituted salicylaldehydes also provided viable starting materials for the synthesis of *meso*-substituted phosphate ester containing BODIPYs **131** with restricted rotations (Scheme 35) [111]. Not surprisingly, due to the lack of conjugation between the BODIPY core and the phenyl substituent in the *meso*-position, the photophysical properties of these dyes appeared largely independent of the nature of the substituents on the phenyl ring (i.e., λ_abs_^max^/λ_em_^max^ 505 nm/520–525 nm, τ 4–6 ns, Φ_f_ 0.3–0.5). This approach provided a convenient platform for studying rotor-like behavior as well as energy and electron transfer processes in nerve agent–simulant systems.

**Scheme 36:**
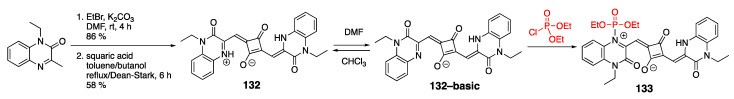
Synthesis of **132** (first step); solvent-controlled equilibrium (second step); tentatively proposed DCP-sensing mechanism by **132–basic** (third step).

Several examples of squaraine-based sensors for detecting phosphorus nerve agents have recently been reported. Squaraine dye **132** showed a chromophoric response in the presence of DCP (Scheme 36) [112]. To follow, **132** was synthesized using a standard approach and revealed solvatochromic behavior (i.e., λ_abs_^max^ (DMF) 621 nm; λ_abs_^max^ (CHCl_3_) 813 nm), which closely resembled pH-dependent changes observed in a related system [113].

The solvent-induced equilibrium between basic and zwitterionic forms of **132** was proposed as an explanation of its behavior. This hypothesis may be reasonable, given the acidic nature of CHCl_3_ and the basic nature of DMF (attributed to DMF’s affinity to complex formation with some Lewis acids). However, since different morphologies were observed by SEM, it could also be plausible that the solvent simply controlled the aggregation and/or solvation state of **132**, resulting in distinct spectral changes. Upon addition of DCP to **132** in DMF, λ_abs_^max^ underwent a shift from 621 nm to 807 nm, with a clear isosbestic point at ca. 670 nm. This provided a relatively straightforward chromophoric, naked-eye detection method for DCP. It was suggested that the nucleophilic nitrogen atom in dye **132** was reactive enough to interact with DCP to give **133**, drawing some parallels to **125**–**127** BODIPY-based dosimeters. It should be noted that similar changes were observed in the presence of HCl, whereas no changes took place upon addition of other simulants, such as DCNP.

**Scheme 37:**
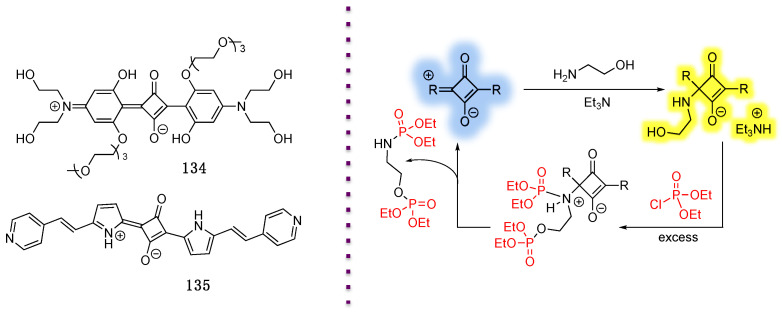
Structures of squaraine dyes **134** and **135** and the tentative sensing mechanism of DCP. Blue and yellow glowing indicates emissive and non-emissive species, respectively.

The electrophilic character of the central four-membered ring in dyes **134** and **135** was exploited by tethering ethanolamine to the squaraine structure, allowing the primary alcohol group to react with the analyte (Scheme 37) [114]. Upon the addition of ethanolamine (330 eq.), the absorption maximum at ca. 600 nm decreased, while the absorption band appeared at ca. 300 nm. Limits of detection were in the 3–35 μM range, although mM amounts of simulants were required for visual detections.

#### 4.1.3. Phosphate-Containing BODIPYs as Photocaging Agents

**Scheme 38:**
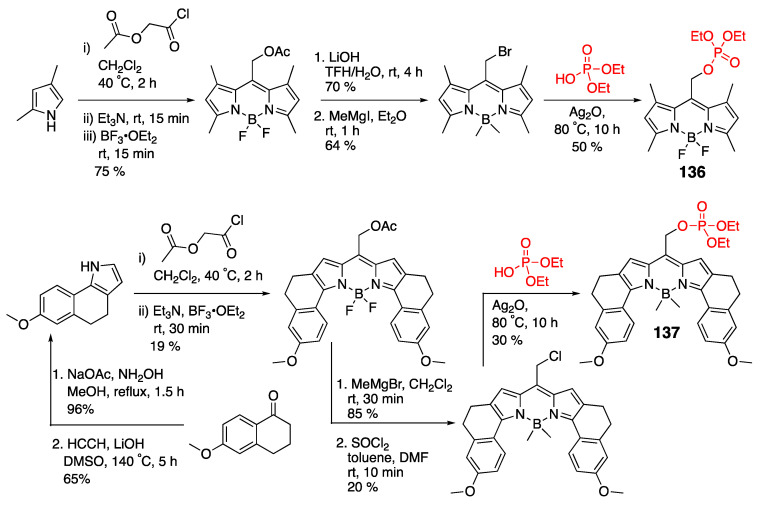
Synthesis of BODIPY photocages **136** and **137**.

Photocages are light-sensitive moieties that temporarily mask the properties of functional groups, which are restorable upon photoinduced removal of the caging moiety. Phosphoesters have been widely employed as viable photocages in a variety of systems as they enable the attachment (and subsequent release) of drugs and probes [115,116]. Due to their photostability, activation across the visible spectral range, and the ability to install functionalities for specific targets, BODIPY dyes have been extensively used as photocaging systems for biological application [117,118]. The phosphate group is a relatively recent addition to the repertoire of BODIPY-based photocaging scaffolds, with only a few examples reported to date (Scheme 38). Photochemical efficiency (i.e., quantum yield multiplied by molar absorptivity) of phosphate-BODIPY photocages **136** and **137** have been shown to be comparable to other linkers, such as ester, halide, carbonate, and carbamate [119].

**Scheme 39:**
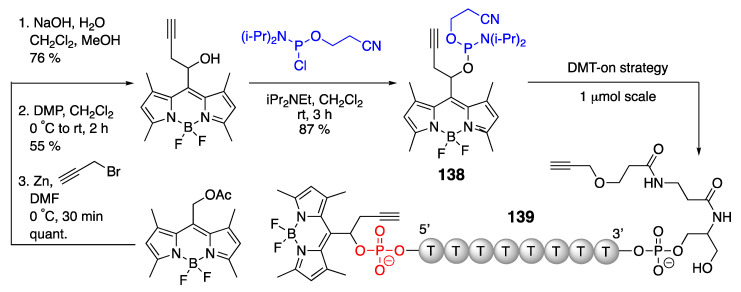
Synthesis of BODIPY-containing oligonucleotide **139**.

BODIPY moiety has been incorporated into an oligonucleotide via solid-state synthesis using phosphoramidite derivative **138**, resulting in **139** (Scheme 39) [120]. Photorelease occurred upon exposure to 530 nm light, demonstrating the potential uses of this system in biologically relevant applications.

#### 4.1.4. Alkylphosphocholine-Containing BODIPY

**Figure 8:**
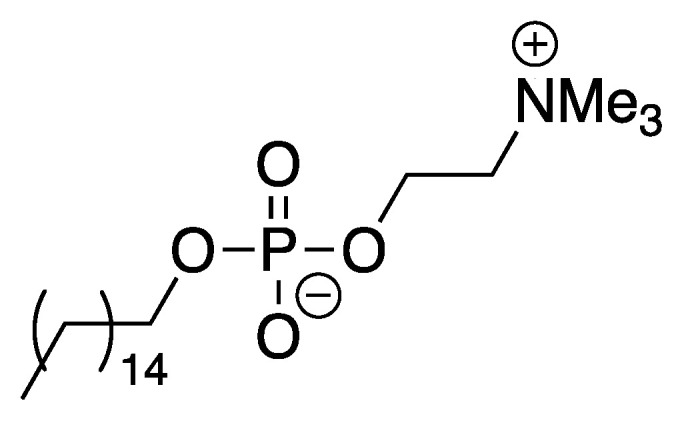
Structure of miltefosine.

The alkylphosphocholine-containing drug miltefosine (Figure 8) has been widely used to treat parasitic diseases and has demonstrated activity against cancer cells, pathogenic bacteria, and fungi [121,122,123].

**Scheme 40:**
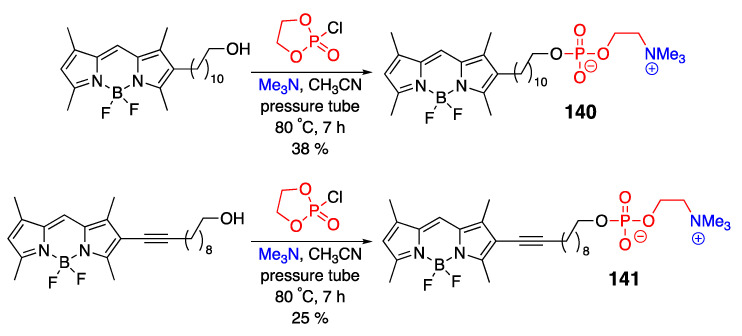
Synthesis of fluorescent miltefosine analogues **140** and **141**.

To better understand miltefosine’s mode of action, several BODIPY-containing imaging agents, i.e., **140** and **141**, have been developed (Scheme 40) [124,125]. These dyes exhibited spectroscopic properties suitable for biological studies and showed specific labeling of the pathogenic areas. Importantly, the introduction of BODIPY moiety did not affect in vitro antiparasitic activity.

**Figure 9:**
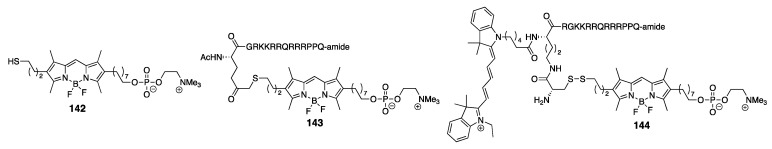
Structure of the sulfide-containing BODIPY dye **142** used for the synthesis of compounds **143** and **144**.

Of particular interest is the thiol-containing analogue **142** (Figure 9), which enabled the tethering of a cell-penetrating peptide to obtain **143** [126]. Additionally, **142** was utilized in the construction of a reduction-sensitive dual-fluorescent system **144** via disulfide bond formation [126].

**Scheme 41:**
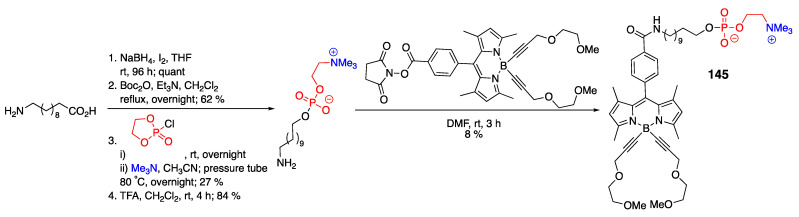
Synthesis of BODIPY **145**.

A more advanced BODIPY motif **145** was developed (Scheme 41), which featured ethylene glycol-containing groups as an effort to avoid aggregation, improve solubility, and enhance brightness in aqueous milieu [127]. This dye effectively stained *Acanthamoeba* trophozite. From the synthesis standpoint, this could be a modular approach enabling access to libraries of fluorescent miltefosine analogues.

### 4.2. Phosphoramidate-Containing BODIPY Dyes

**Scheme 42:**
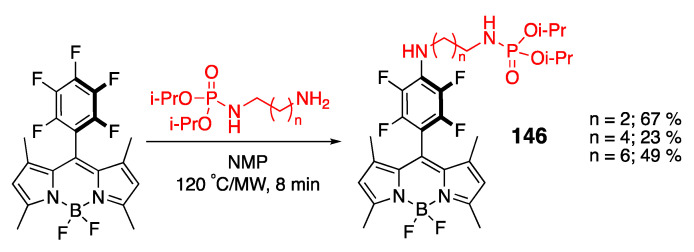
Synthesis of phosphoramidate-containing BODIPY dyes **146**. MW: microwave heating.

Phosphoramidates have been studied for their potential applications in biological systems, such as enzymatic inhibition, anticancer activity, and antiviral effects, as well as for their flame-retardant and anticorrosion properties [128,129,130]. Incorporation of phosphoramidate functionality, i.e., dye **146**, on the BODIPIY scaffold was achieved via nucleophilic aromatic substitution reaction under MW-irradiation heating (Scheme 42) [131]. As expected, the length of the tether between the BODIPY and phosphoramidate functionalities did not impact λ_abs_^max^/λ_em_^max^. However, the emission properties of **146** exhibited a pronounced dependence on solvent polarity: in non-polar solvents (e.g., hexane, THF, and EtOAc), Φ_f_ of 0.82–0.97 were observed, whereas in polar solvents (EtOH, CH_3_CN), Φ_f_ were in the range of 0.03–0.08. This, however, was attributed to the PET process, facilitated by the non-bonding electrons on the nitrogen on the tetrafluoro-substituted aromatic ring in the *meso*-position of the BODIPY core.

**Scheme 43:**
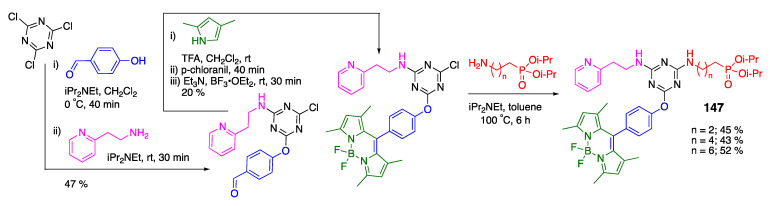
Synthesis of phoshoramidate-containing BODIPY dyes **147**. TFA: trifluoroacetic acid.

Similarly, nucleophilic aromatic substitution on cyanuric chloride was utilized to synthesize **147** (Scheme 43) [132]. Extensive experimentation was required to determine the optimum sequence of introducing substituents, including the synthesis of BODIPY moiety being performed on the cyanuric scaffold. NMR and computational studies revealed the presence of several rotational isomers. However, photophysical properties of these dyes have not yet been reported.

### 4.3. N–P-Containing Dyes: PODIPYs and PHODIPY

**Scheme 44:**
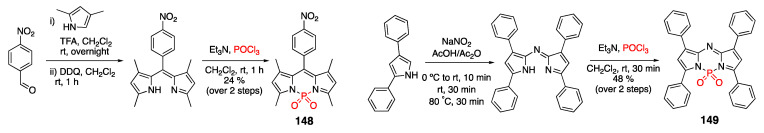
Synthesis of PODIPY dyes **148** and **149**. DDQ: 2,3-dichloro-5,6-dicyano-p-benzoquinone, TFA: trifluoroacetic acid.

BF_2_ moiety is the most prevalent component of dipyrromethene scaffold, although other elements (e.g., Al, Ga, Co, Ni, Fe, Si, Sn, etc.) have also been employed [133,134]. Yet these systems typically exhibit lower stabilities to air and moisture when compared to BODIPYs. Unlike these cases, the incorporation of phosphorus (in the form of PO_2_ moiety) substantially increases the water solubility of the corresponding dyes (so-called PODIPY dyes), especially compared to their BF_2_ analogues. PODIPYs **148** and **149** were synthesized simply by substituting BF_3_·OEt_2_ with POCl_3_, followed by hydrolysis as the last step of scaffold assembly (Scheme 44) [135]. Compound **148** demonstrated some Hg^2+^-sensing capability [136]. Enabling calorimetric detection in solution as well as solid-state was demonstrated (though, regretfully, emission characteristics were not reported).

**Figure 10:**
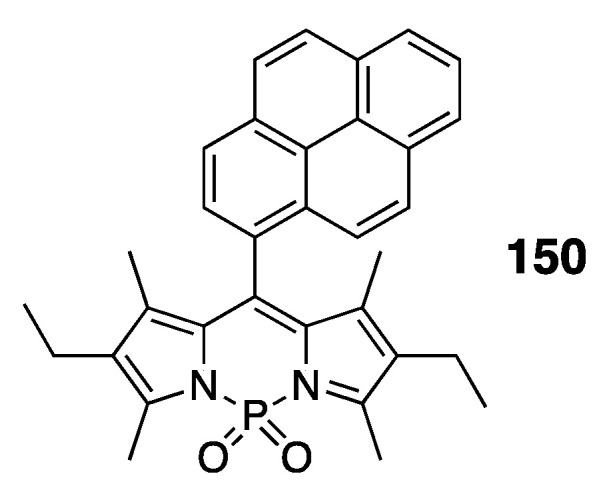
Structure of PODIPY **150**.

Dye **150** (Figure 10) was partially soluble in water, yet it exhibited a complex solvatochromic behavior in organic solvents [137]. However, this dye retained emission in the thin films, suggesting potential solid-state applications [137].

**Figure 11:**
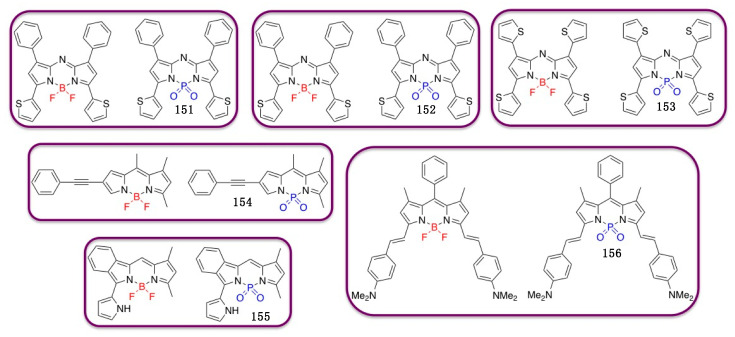
Computational comparison PODIPY **151**–**156** and the corresponding BODIPY dyes.

These promising results prompted a computational study [138] investigating the properties of a more diverse range of PODIPY dyes **151**–**156** (Figure 11). The study utilized a hybrid protocol, which combined the M06-2X functional to calculate ground- and excited-state geometries and vibrational modes, with SOS-CIS(D) to compute transition energies and photophysical properties (e.g., absorption, emission, and adiabatic energies). This wavefunction-based method, complementary to DFT and TD-DFT, is particularly effective for systems with significant charge transfer or strong correlation effects, enabling a more accurate depiction of the electronic transitions and excited-state dynamics.

The study revealed that replacing the BF_2_ group with the PO_2_ group significantly influenced emission energy, dipole moments, and water solvation/complexation. Thus, these observations could have immediate practical implications. Specifically, incorporation of the PO_2_ group was shown to lead to larger Stokes shifts. PODIPYs possess substantially larger dipole moments, which leads to increased solubility of these dyes in polar solvents, including water. These findings highlight the potential of PODIPYs in biological environments where solubility and tunable photophysical properties are crucial.

Although a limited number of PODIPY dyes has ben synthesized to date, this theoretical investigation underscores the versatility of the PO_2_ group as a design component for fine-tuning (photo)physical properties of dipyrromethene dyes. The ability to manipulate key characteristics, such as solubility, emission energy, and charge transfer, makes PODIPYs a promising platform for further exploration in diverse applications.

### 4.4. Cyclotriphosphazene- and Cyclotetraphosphazene-Containing BODIPY Dyes

**Scheme 45:**
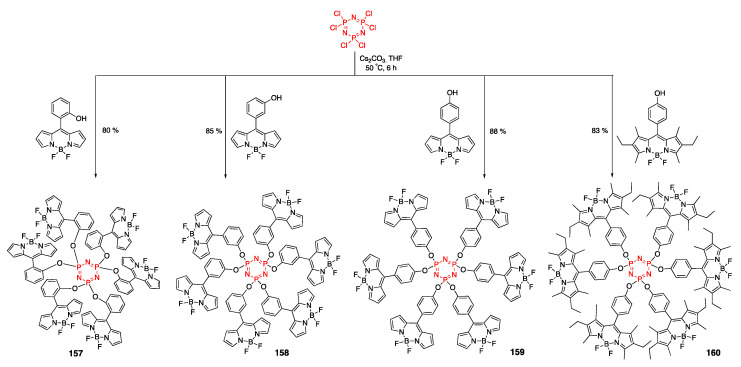
Synthesis of cyclotriphosphazene-containing BODIPYs **157**–**160**.

Cyclotriphosphazene [139,140,141] is an inorganic heterocyclic scaffold that allows for a fairly straightforward incorporation of various chromophoric and fluorophoric units to generate multi-dye systems of broad utility [142,143]. A series of isomeric BODIPY-cyclotriphosphazenes, **157**–**160** (Scheme 45), were prepared by directly reacting the corresponding hydroxy-containing BODIPYs with hexachlorocyclotriphosphazene in the presence of Cs_2_CO_3_ as a base [144]. When compared to the monomeric BODIPYs, these multi-BODIPY scaffolds possessed a number of unique features: ca. three-fold larger extinction coefficients, thus indicating higher brightness; larger Stokes shifts, which suggesting potential application in labeling; and similar quantum yields, which is indicative of all BODIPY units in these dyes behaving independently (although some interaction between BODIPYs were noted in the case of **157** and **158**, based on ^1^H NMR studies).

**Figure 12:**
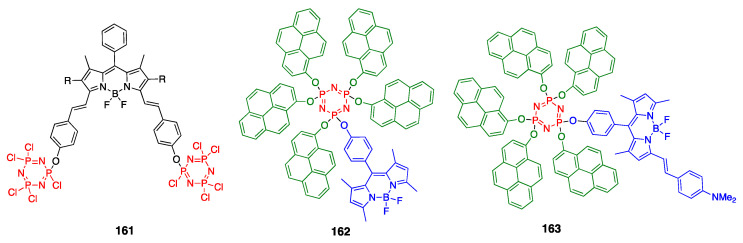
Structures of di-cyclotriphosphazene BODIPY **161** and pyrene-BODIPY dyes **162** and **163**.

The reactivity of the phenolic group was utilized to prepare several BODIPY-bridged di-cyclotriphosphazene dye systems (Figure 12) [145]. **161-H**, **161-Br**, and **161-I** virtually retained the photophysical characteristics as the parent BODIPY dyes (λ_abs_^max^/λ_em_^max^, and τ) while exhibiting ca. two-fold higher extinction coefficients. Not surprisingly, the presence of Br and I atoms in **161-Br** and **161-I** resulted in systems capable of singlet oxygen generation, indicating that these compounds could be used as photosensitizers.

Heterofluorophoric systems, featuring BODIPY and pyrene units, i.e., **162** and **163**, were also developed (Figure 12) to evaluate Förster resonance energy transfer (FRET) [146]. Both **162** and **163** dyes showed that a significant quenching of the pyrene’s (donor) emission was accompanied by enhanced emission of the BODIPY (acceptor) upon excitation of pyrene, thus indicating a very efficient FRET. The large Stokes shifts stemming from well-separated absorption and emission of pyrene and BODIPY units make these systems attractive for (bio)imaging applications, albeit structural modifications might be required to increase solubility in aqueous milieu, along with shifting excitation wavelength into the red region [146].

**Figure 13:**
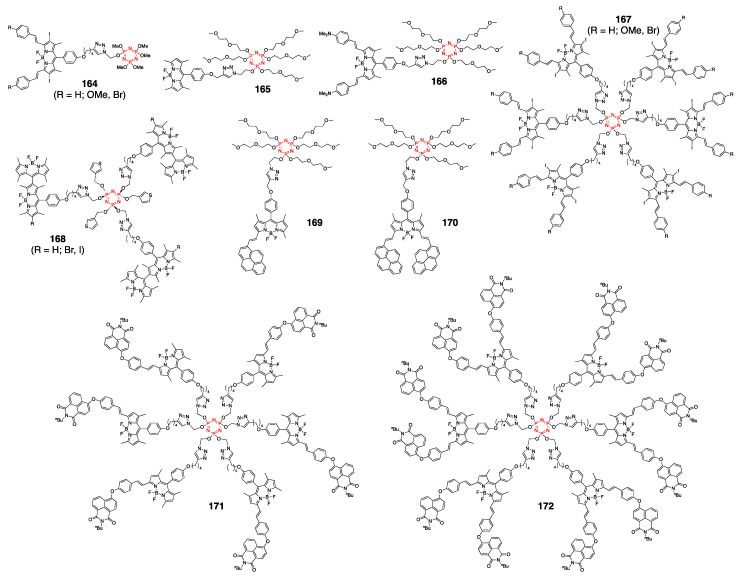
Structures of BODIPY-containing cyclotriphosphazene-based dyes **164**–**172**; citation is given in brackets next to the compound number: **164** [147], **165** [148], **166** [ 148], **167** [149], **168** [150], **169** [151], **170** [151], **171** [152], **172** [152].

Using copper-catalyzed alkyne-azide cycloaddition (CuAAC, so-called click-chemistry), multi-BODIPY systems, such as pyrene–BODIPY and napthalimide–BODIPY dyads, were grafted onto a cyclotriphosphazene scaffold to give dyes **164**–**172** (Figure 13) [147,148,149,150,151,152]. Additionally, incorporation of ethylene glycol moieties further enhanced the water solubility. Photophysical properties of these poly-dye systems were relatively like the individual dyes. However, in some systems, solvatochromic behavior was observed [151]. For example, **170** (Figure 13) displayed a pronounced solvatochromic effect, with λ_em_^max^ varying within the 691–711 nm range, although **170** was not emissive in water, EtOH, and hexane. In contrast, **169** showed negligible solvatochromism across solvents of different polarity (i.e., water, acetone, dichloromethane, and hexane): λ_abs_^max^/λ_em_^max^ 497–500 nm/508–510 nm) [151].

**Figure 14:**
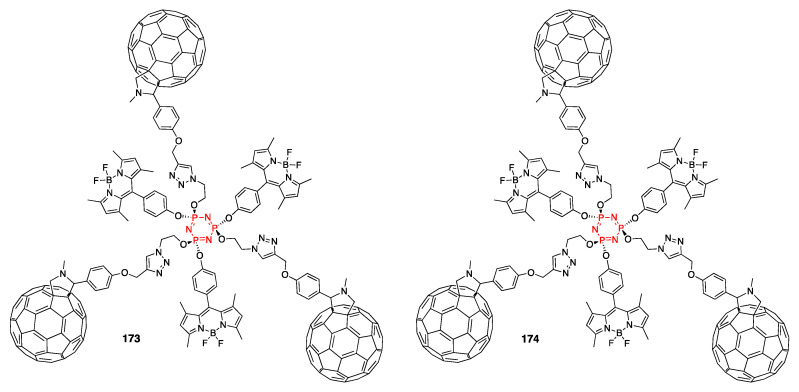
Structures of fullerene-containing BODIPYs **173** and **174** on a cyclotriphosphazene scaffold.

More elaborate, three-component assemblies, such as BODIPY–cyclotriphosphazene–fullerene–C_60_ **173** and **174** (Figure 14), were also prepared [153]. Though **173** and **174** were good systems to study energy/electron transfer processes, their photophysical and photochemical properties (i.e., ^1^O_2_ generation) properties were found to be similar or even inferior to non-fullerene-containing assemblies.

**Figure 15:**
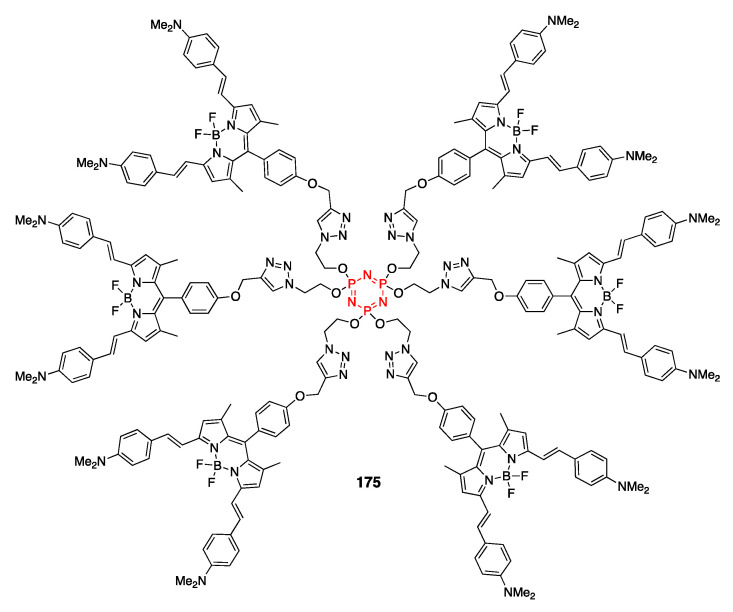
Structure of **175** used as a theranostic agent.

Encapsulation of **175** (Figure 15) [154] with an amphiphilic polymer (e.g., DSPE-PEG2000 or PEG 5000-PLA3000) produced water-soluble nanoparticles (NPs, with an average size of ca. 90 nm), with potential theranostic applications [155]. Specifically, photoacoustic and fluorescence imaging, along with photothermal treatment, were demonstrated. Notably, **175-NP** selectively accumulated in 4T1 tumor-bearing mice, effectively suppressing tumor size upon exposure to 655 nm laser light [155].

**Scheme 46:**
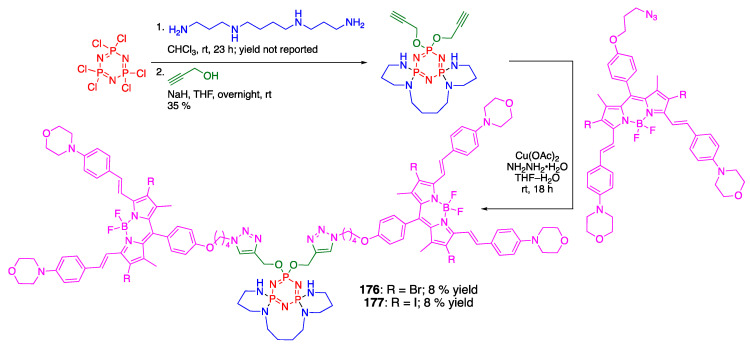
Synthesis of spermine-modified cyclotriphosphazene BODIPYs **176** and **177**.

Recently, spermine-modified cyclotriphosphazene scaffold was functionalized using styryl-containing BODIPY dyes (Scheme 46) [156]. Dyes **176** and **177** exhibited strong absorption and emission in the near-infrared region (i.e., λ_abs_^max^/λ_em_^max^ ca. 715 nm/815 nm), high photostability, and efficient singlet oxygen generation. However, all photophysical and photochemical properties of these dyes were similar to other cyclotriphosphazine-containing BODIPY dyes (e.g., Figure 13). Notably, however, red-light illumination of these halogenated derivatives induced selective phototoxicity, effectively targeting PC-3 cancer cells [156].

**Figure 16:**
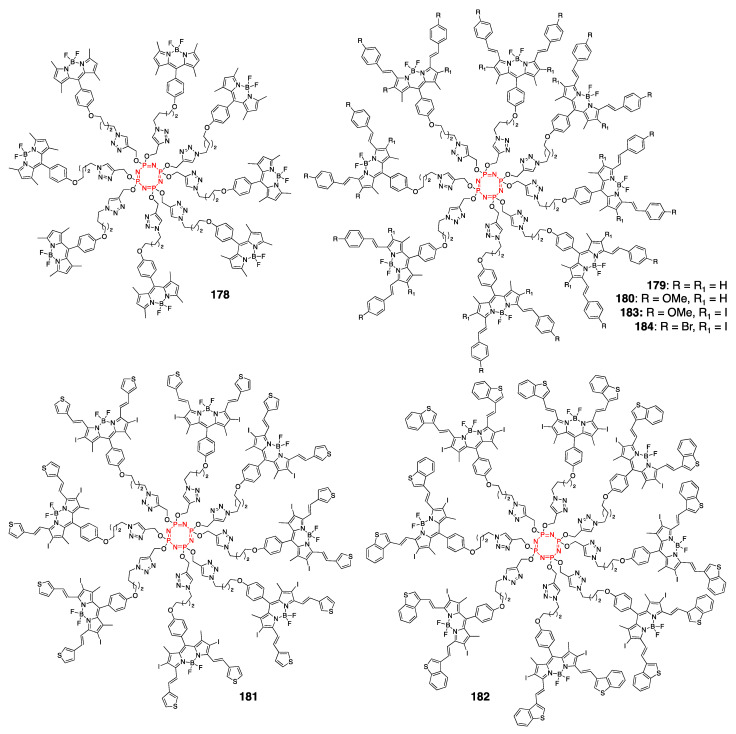
Structures of cyclotetraphosphazene-containing BODIPY dyes **178**–**184**.

Several studies exploited cyclotetraphosphazene scaffold, a less commonly utilized analogue of cyclotriphosphazene to construct BODIPY-containing systems **178**–**182** as ON-OFF cation sensors [157,158]. For example, **179** and **180** displayed high selectivity toward Co^2+^ over other transition metals (e.g., Cr^3+^, Fe^3+^, Cu^2+^, Fe^2+^, Mn^2+^, Cd^2+^, Ag^+^, and Hg^+^) as well as alkali and alkali earth cations (e.g., Li^+^, Na^+^, K^+^, Mg^2+^, Ca^2+^, and Ba^2+^). It is of interest to point that the incorporation of thiophene and thianaphthene moieties onto BODIPY produced ON-OFF sensors **181** and **182** (Figure 16) that exhibited selectivity not only toward Co^2+^ but also toward Cu^2+^, albeit with inferior sensitivity and detection limits. Interestingly, the extinction coefficients of **181** and **182** were substantially larger than those of **178**–**180**, thus indicating that the incorporation of some groups onto BODIPY–cyclotetraphosphazenes could potentially lead to the development of high brightness materials. However, further optimization of these structures might be needed to allow for practical utilization in metal-sensing applications due to relatively low (a) sensitivity levels (e.g., only ca. two-fold decrease of fluorescence intensity of **181** was observed in the presence of 500 molar excess of Co^2+^) and (b) detection limits (low μM range).

BODIPY-functionalized cyclotetraphosphazines **183** and **184** were additionally investigated as potential photosensitizers (Figure 16) [159]. However, increasing the number of BODIPY units from six to eight did not significantly alter the photochemical properties or singlet oxygen-generating capability, emphasizing the challenges in developing robust structure–property relationships for polyfluorophoric systems.

## 5. Conclusions

In this review, we attempted to summarize available data on phosphorus-containing BODIPY and squaraine dyes. To demonstrate the versatility of phosphorus-based moieties in tuning various photochemical properties, this review highlighted their roles in enhancing solubility, modulating emission energies, enabling efficient energy and electron transfer processes, and facilitating the design of multifunctional systems for applications ranging from bioimaging and sensing to theranostics and photodynamic therapy. While the incorporation of phosphorus groups does not always result in a unique set of photophysical properties, their versatility enables the creation of fluorogenic scaffolds with expanded functionality and application potential.

Despite these advantages, limitations remain. The stability of many P-containing dyes is a significant concern, as it could lead to rapid degradation and reduced photostability, consequently hindering their practical utility. Furthermore, the tendency of some P-containing dyes to aggregate in aqueous environments could compromise imaging applications due to aggregation-induced quenching, a common challenge in organic fluorophores, particularly in biological contexts [160,161].

Considering the broad diversity of P-containing functional groups, ongoing advancements in the development of novel fluorogenic materials tailored for emerging and advanced applications are highly anticipated. Advances in aggregation-induced emission luminogens provide a promising approach to overcoming fluorescence quenching issues, allowing to expand the utility of these materials in imaging and sensing applications [160,161]. Similarly, advancements in lipid droplet-localized photosensitizers offer valuable insights into targeting specific microenvironments [162,163]. By leveraging current progress, ongoing efforts can aim to refine P-containing dyes for broader and more versatile applications across bioimaging, sensing, and theranostics.

## Data Availability

Not applicable.
